# Supplementary figures and images for: Efficacy and safety of electrical acupoint stimulation for postoperative nausea and vomiting: A systematic review and meta-analysis
Source: PLoS One. 2023 May 31;18(5):e0285943. doi: 10.1371/journal.pone.0285943 (PMC10231798; doi:10.1371/journal.pone.0285943)

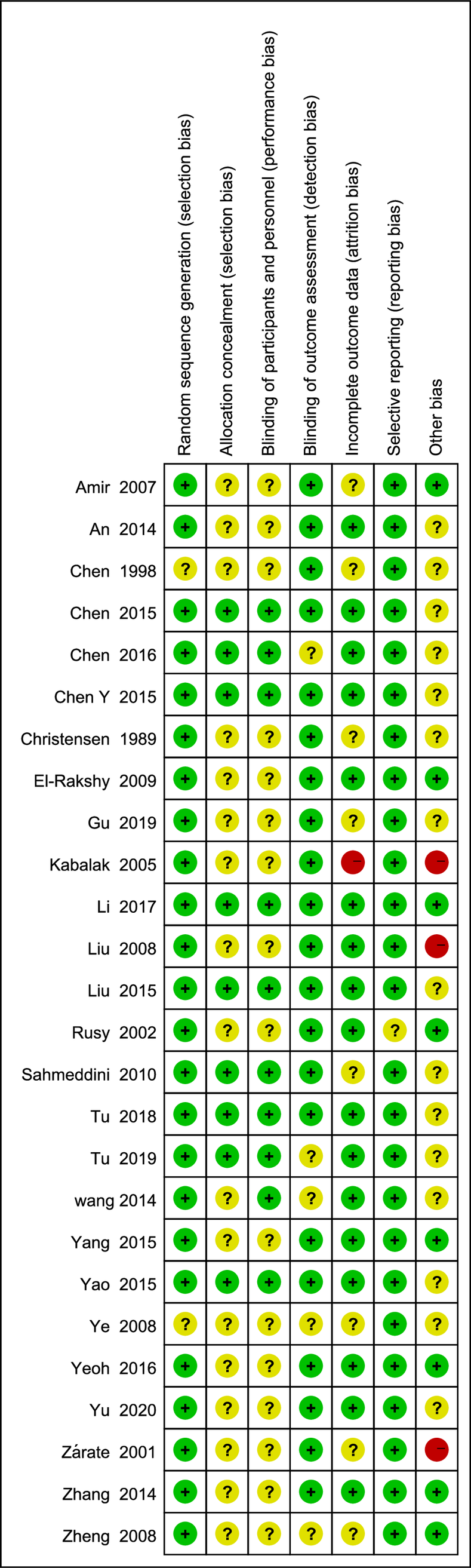

Supplement: S1 Fig — (TIF) [file pone.0285943.s002.tif]

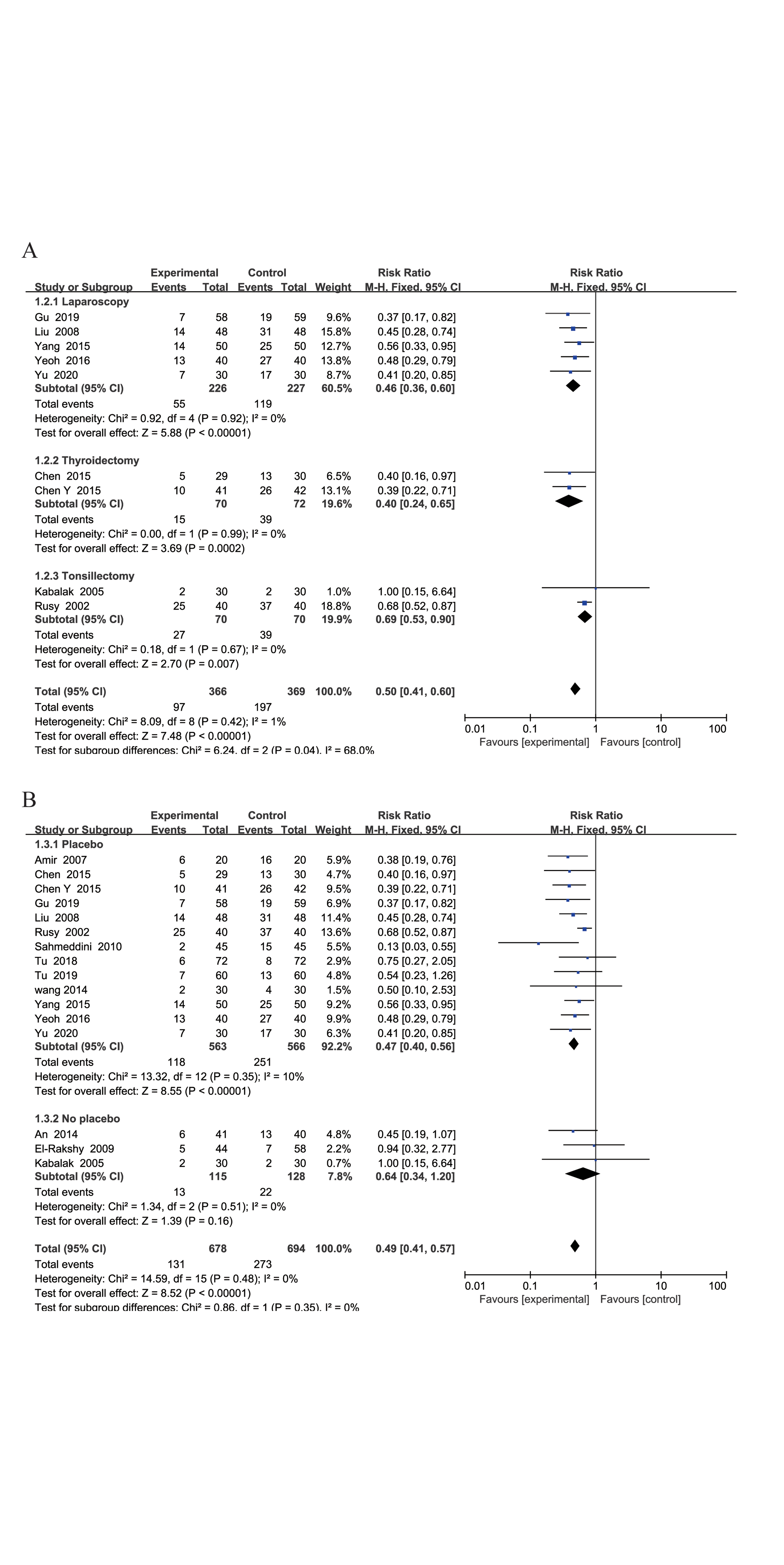

Supplement: S2 Fig — A: Forest plots of types of surgical procedures. B: Forest plot of EAS vs. placebo. (TIF) [file pone.0285943.s003.tif]

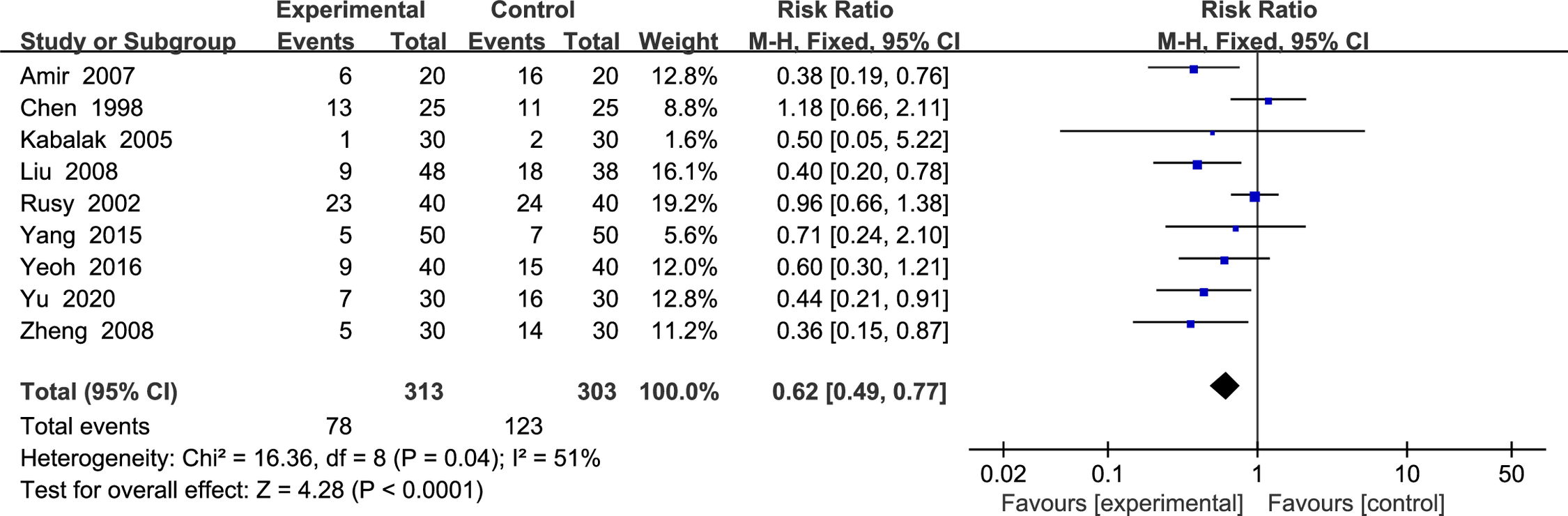

Supplement: S3 Fig — (TIF) [file pone.0285943.s004.tif]

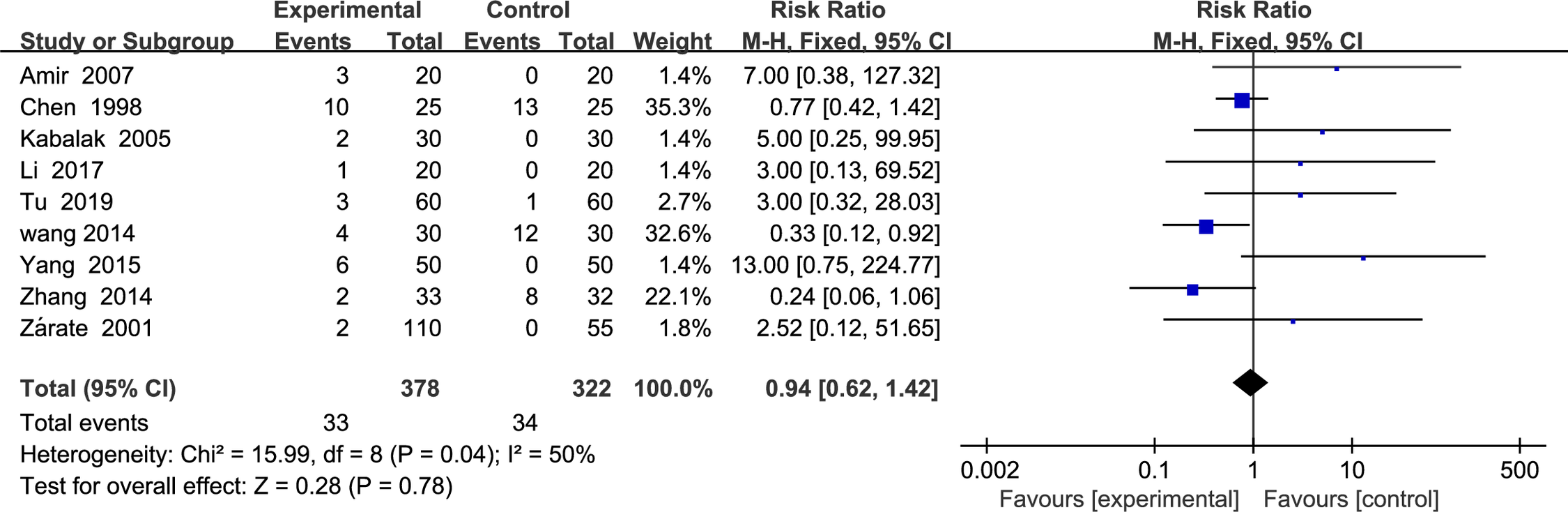

Supplement: S4 Fig — (TIF) [file pone.0285943.s005.tif]

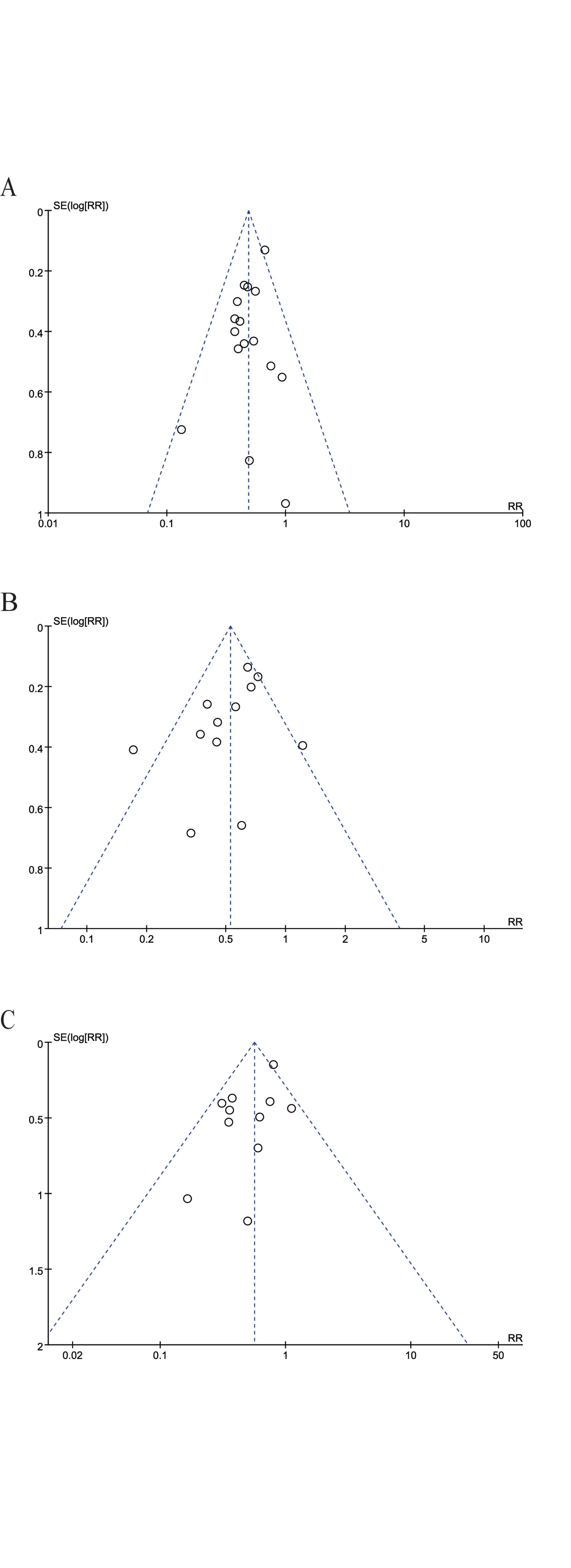

Supplement: S5 Fig — A: Funnel plot for postoperative nausea and vomiting. B: Funnel plot for postoperative nausea. C: Funnel plot for postoperative vomiting. (TIF) [file pone.0285943.s006.tif]
