# Supplementary material for: Efficacy and safety of electrical acupoint stimulation for postoperative nausea and vomiting: A systematic review and meta-analysis
Source: PLoS One. 2023 May 31;18(5):e0285943. doi: 10.1371/journal.pone.0285943 (PMC10231798; doi:10.1371/journal.pone.0285943)
Supplement: S1 Table — (DOCX) [file pone.0285943.s007.docx]

**S1 Table. Search strategy**

1. **The PubMed database**
2. Postoperative Nausea and Vomiting [Mesh]
3. (“post operative” OR “postoperati*” OR “perioperati*” OR “peri-operative” OR “surger*” OR “surgical*” OR “postsurg*” OR “intraoperative” OR “anesthe*” OR “anaesthe*” OR “postanesthe*” OR “postanaesthe*” OR “anaesthetic recovery”)
4. (“nause*” OR “vomit*” OR “emesis” OR “emeses” OR “emet*” OR “queasiness” OR “queasy”)
5. #2 AND #3
6. #1 OR #4
7. Electroacupuncture [Mesh]
8. electric*
9. (acupuncture OR needle OR acupoint OR point OR stimulat*)
10. #7 AND #8
11. #6 OR #9
12. Transcutaneous Electric Nerve Stimulation [Mesh]
13. (“Transcutaneous Electrical Acupoint Stimulation” OR “TENS” OR “TEAS” OR “TNS” OR “ENS” OR “TES” OR “Transcutaneous electric* nerve stimulation” OR “transcutaneous nerve stimulation” OR “transcutaneous electric*” OR “transcutaneous electric* stimulation” OR “electric* nerve therap*” OR “electroanalgesi*” OR “electro-analgesi*” OR “Percutaneous Electric*” OR “Percutaneous Neuromodulation therap*” OR “Electroanalgesia*” OR “nerve stimulat*” OR “neuro-modulation” OR “neuromodulation” OR “neuromusc* electric*”)
14. #11 OR #12
15. Electric Stimulation Therapy [Mesh]
16. (“Electric Stimulation Therapy” OR “Electric* Stimulation” OR “electrotherap*” OR “electrostimul*” OR “electromyostimulation” OR “Interferential Current Electrotherapy” OR “Therapeutic Electric* stimulat*” OR “Electric* stimulat* therap*”)
17. #14 OR #15
18. (“random* controlled trial” OR “random*” OR “placebo”)]
19. #10 OR #13 OR #16
20. #5 AND #18 AND #17
21. **The** **Cochrane Library database**
22. MeSH descriptor: [Postoperative Nausea and Vomiting] explode all trees
23. ("post operative"):ti,ab,kw OR (postoperati*):ti,ab,kw OR (perioperati*):ti,ab,kw OR (peri-operative):ti,ab,kw OR (surger*):ti,ab,kw OR (surgical*):ti,ab,kw OR (postsurg*):ti,ab,kw OR (intraoperative):ti,ab,kw OR (anesthe*):ti,ab,kw OR (anaesthe*):ti,ab,kw OR (postanesthe*):ti,ab,kw OR (postanaesthe*):ti,ab,kw OR (anaesthetic recovery):ti,ab,kw
24. (nause*):ti,ab,kw OR (vomit*):ti,ab,kw OR (emesis):ti,ab,kw OR (emeses):ti,ab,kw OR (emet*):ti,ab,kw OR (queasiness):ti,ab,kw OR (queasy):ti,ab,kw
25. (#2 AND #3) OR #1
26. MeSH descriptor: [Electroacupuncture] explode all trees
27. ((electric*):ti,ab,kw) AND ((acupuncture):ti,ab,kw OR (needle):ti,ab,kw OR (acupoint):ti,ab,kw OR (stimulat*):ti,ab,kw) OR (“electrical acupuncture”):ti,ab,kw OR (electro-acupuncture):ti,ab,kw
28. #5 OR #6
29. MeSH descriptor: [Electric Stimulation Therapy] explode all trees
30. MeSH descriptor: [Transcutaneous Electric Nerve Stimulation] explode all trees
31. (“TENS”):ti,ab,kw OR (“TNS”):ti,ab,kw OR (“ENS”):ti,ab,kw OR (“TES”):ti,ab,kw OR (“Transcutaneous electric* nerve stimulation”):ti,ab,kw OR (“transcutaneous nerve stimulation”):ti,ab,kw OR ("transcutaneous electric*"):ti,ab,kw OR (“transcutaneous electric* stimulation”):ti,ab,kw OR ("electric* nerve therap*"):ti,ab,kw OR (electroanalgesi*):ti,ab,kw OR (electro-analgesi*):ti,ab,kw OR ("Percutaneous Electric*"):ti,ab,kw OR ("Percutaneous Neuromodulation therap*"):ti,ab,kw OR (Electroanalgesia*):ti,ab,kw OR (“nerve stimulat*”):ti,ab,kw OR (neuro-modulation):ti,ab,kw OR (neuromodulation):ti,ab,kw OR (“neuromusc* electric*”):ti,ab,kw

#11. #9 OR #10

#12. #7 OR #8 OR #11

#13. #4 AND #12

1. **The** **Embase database**
2. 'postoperative nausea and vomiting'/exp
3. 'postoperative complications':ti,ab
4. 'postoperative complication':ti,ab
5. 'ponv':ti,ab
6. 'nausea and vomiting, postoperative':ti,ab
7. 'vomiting, postoperative':ti,ab
8. 'postoperative emesis':ti,ab
9. 'postoperative vomiting':ti,ab
10. 'emesis, postoperative':ti,ab
11. 'emeses, postoperative':ti,ab
12. 'postoperative emeses':ti,ab
13. 'postoperative nausea':ti,ab
14. 'nausea, postoperative':ti,ab
15. #1 OR #2 OR #3 OR #4 OR #5 OR #6 OR #7 OR #8 OR #9 OR #10 OR #11 OR #12 OR #13
16. 'postoperative period'/exp
17. 'postoperative periods':ti,ab
18. 'post operative':ti,ab
19. 'postoperative':ti,ab
20. 'peri-operative':ti,ab
21. 'period, postoperative':ti,ab
22. 'periods, postoperative':ti,ab
23. #15 OR #16 OR #17 OR #18 OR #19 OR #20 OR #21
24. 'nausea'/exp
25. 'emesis':ti,ab
26. 'emeses':ti,ab
27. 'vomiting':ti,ab
28. 'queasiness':ti,ab
29. 'queasy':ti,ab
30. #23 OR #24 OR #25 OR #26 OR #27 OR #28
31. #22 AND #29
32. #14 OR #30
33. #31. #14 OR #30
34. 'electrical acupuncture':ti,ab
35. 'electro-acupuncture':ti,ab
36. 'electric':ti,ab
37. 'electrical':ti,ab
38. 'acupuncture':ti,ab
39. 'needle':ti,ab
40. 'acupoint':ti,ab
41. 'point':ti,ab
42. 'stimulat':ti,ab
43. #35 OR #36
44. #37 OR #38 OR #39 OR #40 OR #41
45. #42 AND #43
46. #32 OR #33 OR #34 OR #44
47. 'electrotherapy'/exp
48. 'electric stimulation':ti,ab
49. 'electrical acustimulation':ti,ab
50. 'therapeutic electrical stimulation':ti,ab
51. 'electrical stimulation, therapeutic':ti,ab
52. 'stimulation, therapeutic electrical':ti,ab
53. 'therapeutic electric stimulation':ti,ab
54. 'electric stimulation, therapeutic':ti,ab
55. 'stimulation, therapeutic electric':ti,ab
56. 'electrical stimulation therapy':ti,ab
57. 'stimulation therapy, electrical':ti,ab
58. 'therapy, electrical stimulation':ti,ab
59. 'therapy, electric stimulation':ti,ab
60. 'stimulation therapy, electric':ti,ab
61. 'electrotherapy':ti,ab
62. 'interferential current electrotherapy':ti,ab
63. 'electrotherapy, interferential current':ti,ab
64. #46 OR #47 OR #48 OR #49 OR #50 OR #51 OR #52 OR #53 OR #54 OR #55 OR #56 OR

#57 OR #58 OR #59 OR #60 OR #61 OR #62

1. 'transcutaneous electrical nerve stimulation'/exp
2. 'electric stimulation, transcutaneous':ti,ab
3. 'stimulation, transcutaneous electric':ti,ab
4. 'stimulation, transcutaneous electric':ti,ab
5. 'percutaneous electric nerve stimulation':ti,ab
6. 'tens':ti,ab
7. 'electrical stimulation, transcutaneous':ti,ab
8. 'transcutaneous electrical stimulation':ti,ab
9. 'transdermal electrostimulation':ti,ab
10. 'electrostimulation, transdermal':ti,ab
11. 'percutaneous electrical nerve stimulation':ti,ab
12. 'transcutaneous electrical nerve stimulation':ti,ab
13. 'transcutaneous nerve stimulation':ti,ab
14. 'nerve stimulation, transcutaneous':ti,ab
15. 'stimulation, transcutaneous nerve':ti,ab
16. 'percutaneous neuromodulation therapy':ti,ab
17. 'neuromodulation therapy, percutaneous':ti,ab
18. 'percutaneous neuromodulation therapies':ti,ab
19. 'therapy, percutaneous neuromodulation':ti,ab
20. 'percutaneous electrical neuromodulation':ti,ab
21. 'electrical neuromodulation, percutaneous':ti,ab
22. 'electrical neuromodulations, percutaneous':ti,ab
23. 'neuromodulation, percutaneous electrical':ti,ab
24. 'neuromodulations, percutaneous electrical':ti,ab
25. 'percutaneous electrical neuromodulations':ti,ab
26. 'analgesic cutaneous electrostimulation':ti,ab
27. 'cutaneous electrostimulation, analgesic':ti,ab
28. 'electrostimulation, analgesic cutaneous':ti,ab
29. 'electroanalgesia':ti,ab
30. 'electroanalgesias':ti,ab
31. #64 OR #65 OR #66 OR #67 OR #68 OR #69 OR #70 OR #71 OR #72 OR #73 OR #74 OR

#75 OR #76 OR #77 OR #78 OR #79 OR #80 OR #81 OR #82 OR #83 OR #84 OR #85 OR

#86 OR #87 OR #88 OR #89 OR #90 OR #91 OR #92 OR #93

1. 'random':ti,ab
2. 'placebo':ti,ab
3. 'double-blind':ti,ab
4. #95 OR #96 OR #97
5. #45 OR #63 OR #94

#100. #31 AND #98 AND #99

1. **The** **Web of Science database**
2. (“electric*” AND (“acupuncture” OR “needle” OR “acupoint” OR “point” OR “stimulat*”))
3. (“Electric Stimulation Therapy” OR “Electric* Stimulation” OR “electrotherap*” OR “electrostimul*” OR “electromyostimulation” OR “Interferential Current Electrotherapy” OR “Therapeutic Electric* stimulat*” OR “Electric* stimulat* therap*”)
4. (“Transcutaneous Electric Nerve Stimulation” OR “TENS” OR “TNS” OR “ENS” OR “TES” OR “Transcutaneous electric* nerve stimulation” OR “transcutaneous nerve stimulation” OR “transcutaneous electric*” OR “transcutaneous electric* stimulation” OR “electric* nerve therap*” OR “electroanalgesi*” OR “electro-analgesi*” OR “Percutaneous Electric*” OR “Percutaneous Neuromodulation therap*” OR “Electroanalgesia*” OR “nerve stimulat*” OR “neuro-modulation” OR “neuromodulation” OR “neuromusc* electric*”)
5. (“post operative” OR “postoperati*” OR “perioperati*” OR “peri-operative” OR “surger*” OR “surgical*” OR “postsurg*” OR “intraoperative” OR “anesthe*” OR “anaesthe*” OR “postanesthe*” OR “postanaesthe*” OR “anaesthetic recovery”) AND (“nause*” OR “vomit*” OR “emesis” OR “emeses” OR “emet*” OR “queasiness” OR “queasy”)
6. (“random* controlled trial” OR “random*” OR “placebo”)
7. (#1 OR #2 OR #3) AND #4 AND #5
8. **ClinicalTrials.gov**
9. Status: All studies

Condition or disease: postoperative nausea and vomiting

Other terms: electric

1. Status: All studies

Condition or disease: postoperative nausea and vomiting

Other terms: acupuncture

1. Status: All studies

Condition or disease: postoperative nausea and vomiting

Other terms: needle

1. Status: All studies

Condition or disease: postoperative nausea and vomiting

Other terms: acupoint

1. Status: All studies

Condition or disease: postoperative nausea and vomiting

Other terms: Transcutaneous Electric Nerve Stimulation

1. Status: All studies

Condition or disease: postoperative nausea and vomiting

Other terms: TENS

1. Status: All studies

Condition or disease: postoperative nausea and vomiting

Other terms: TEAS

1. Status: All studies

Condition or disease: postoperative nausea and vomiting

Other terms: Stimulation

1. Status: All studies

Condition or disease: postoperative nausea and vomiting

Other terms: Transcutaneous

1. #1 OR #2 OR #3 OR #4 OR #5 OR #6 OR #7 OR #8 OR #9
