# Supplementary material for: Efficacy and safety of electrical acupoint stimulation for postoperative nausea and vomiting: A systematic review and meta-analysis
Source: PLoS One. 2023 May 31;18(5):e0285943. doi: 10.1371/journal.pone.0285943 (PMC10231798; doi:10.1371/journal.pone.0285943)
Supplement: S2 Table — (DOCX) [file pone.0285943.s008.docx]

**S2 Table. Details of the included trials for treatments methods.**

| Author (year) | Treatment site | Electrical stimulation intensity | Evaluation schedule |
| --- | --- | --- | --- |
| Yeoh et al, 2016[42] | P6 | 5-40 mA | 24 h after surgery |
| Kabalak et al, 2005[43] | P6; CV13 | 20 Hz  10 mA | 24 h after surgery |
| Zárate et al, 2001[44] | P6 | 31 Hz  25 mA | 9 h after surgery |
| Ye et al, 2008[45] | LI4; PC8; HT7; Auriculotherapy | 2/100 Hz  10-20 mA | 24 h after surgery |
| Chen et al, 1998[46] | ST36 | 2/100 Hz  9-12 mA | 72 h after surgery |
| Liu et al, 2008[47] | P6 | 2/100 Hz  0.5-4 mA | 24 h after surgery |
| An et al, 2014[48] | LI4; SJ5; BL63; LR3;  ST36; GB40 | 2/100 Hz | 48 h after surgery |
| Rusy et al, 2002[49] | P6 | 4 Hz | 24 h after surgery |
| Sahmeddini et al, 2010[50] | LI4; LI11; HT7; HC6 | 10/80 Hz  4 mA | 6 h after surgery |
| El-Rakshy et al, 2009[51] | CV2, GV4, BL32, BL23, LI4, PC6, LR3, SP6 | 10 Hz | 24 h after surgery |
| Christensen et al, 1989[52] | CV2, GV4, BL32, SP6 | 10/100 Hz  12 V | 6 h after surgery |
| Zhang et al, 2014[53] | LI4; PC6; ST36 | 2/10 Hz  6-9 mA | 24 h after surgery |
| Tu et al, 2018[54] | BL13; LI4; ST36 | 2/100hz  5-30 mA | During the period of postanesthesia care unit |
| Tu et al, 2019[55] | BL23; SP9 | 2/100 Hz  5-30 mA | 48 h after surgery |
| Li et al, 2017[56] | PC6; ST36 | 20/100 Hz | 24 h after surgery |
| Gu et al, 2019[57] | ST36; PC6 | 2/100 Hz  5-30 mA | 36 h after surgery |
| Amir et al, 2007[58] | P6 | 4 Hz | 24 h after surgery |
| Chen et al, 2016[59] | LR3; GB34; TE5; LU5 | 2 Hz  3-5 mA。 | 72 h after surgery |
| Yang et al, 2015[60] | P6 | 2 Hz  6-20 mA | 24 h after surgery |
| Yu et al, 2020[61] | GV20; EX-HN3; ST36; PC6 | 2/100 Hz  12-15 mA | 24 h after surgery |
| Liu et al, 2015[62] | LI4; TE5; BL63; LR3; ST36; GB 40; GB20; BL10; BL2; EX-HN4 | 2/100 Hz | 72 h after surgery |
| Chen et al, 2015[63] | LI4; PC6 | 2/10 Hz  6-9 mA | 24 h after surgery |
| Chen Y et al, 2015[64] | LI4; PC6 | 2/10 Hz  6-9 mA | 24 h after surgery |
| Yao et al, 2015[65] | LI4; PC6; ST36; SP6 | 2/10 Hz  6-9 mA | 24 h after surgery |
| Zheng et al, 2008[66] | LI4; PC6 | 2/100 Hz  8-30 mA | 24 h after surgery |
| wang et al, 2014[67] | LI4; PC6; ST36 | 2/10 Hz  6-9 mA | 24 h after surgery |
