# Supplementary material for: Efficacy and safety of electrical acupoint stimulation for postoperative nausea and vomiting: A systematic review and meta-analysis
Source: PLoS One. 2023 May 31;18(5):e0285943. doi: 10.1371/journal.pone.0285943 (PMC10231798; doi:10.1371/journal.pone.0285943)
Supplement: S3 Table — (DOCX) [file pone.0285943.s009.docx]

**S3 Table. Sensitivity analyses by excluding one study at a time**

| Outcome | Excluded  Study | Heterogeneity test | | | Effect Model | Meta-analysis results | | |
| --- | --- | --- | --- | --- | --- | --- | --- | --- |
|  |  | *I^2^*-value | | P-value |  | 95% CI | Z-value | *P*-value |
| PONV within 24 | Amir et al, 2007[47] | 0 | = 0.63 | | Fixed | 0.52 [0.43, 0.62] | 7.13 | ＜0.00001 |
|  | An et al, 2014[37] | 0 | = 0.54 | | Fixed | 0.51 [0.43, 0.61] | 7.39 | ＜0.00001 |
|  | Chen et al, 2015[52] | 0 | = 0.58 | | Fixed | 0.51 [0.43, 0.61] | 7.32 | ＜0.00001 |
|  | Chen Y et al, 2015[53] | 0 | = 0.65 | | Fixed | 0.52 [0.43, 0.63] | 6.94 | ＜0.00001 |
|  | El-Rakshy et al, 2009[40] | 0 | = 0.60 | | Fixed | 0.49 [0.41, 0.59] | 7.78 | ＜0.00001 |
|  | Kabalak et al, 2005[32] | 0 | = 0.55 | | Fixed | 0.50 [0.42, 0.60] | 7.67 | ＜0.00001 |
|  | Liu et al, 2008[36] | 0 | = 0.58 | | Fixed | 0.52 [0.43, 0.62] | 6.90 | ＜0.00001 |
|  | Rusy et al, 2002[38] | 0 | = 0.96 | | Fixed | 0.47 [0.38, 0.58] | 7.01 | ＜0.00001 |
|  | wang et al, 2014[56] | 0 | = 0.52 | | Fixed | 0.51 [0.43, 0.60] | 7.56 | ＜0.00001 |
|  | Yang et al, 2015[49] | 0 | = 0.51 | | Fixed | 0.50 [0.42, 0.60] | 7.31 | ＜0.00001 |
|  | Yeoh et al, 2016[31] | 0 | = 0.54 | | Fixed | 0.51 [0.42, 0.62] | 7.03 | ＜0.00001 |
|  | Yu et al, 2020[50] | 0 | = 0.59 | | Fixed | 0.51 [0.43, 0.62] | 7.20 | ＜0.00001 |
| PONV at other time | Gu et al, 2019[46] | 52 | = 0.13 | | Fixed | 0.42 [0.24, 0.74] | 3.02 | = 0.003 |
|  | Sahmeddini et al, 2010[39] | 0 | = 0.56 | | Fixed | 0.50 [0.31, 0.83] | 2.71 | = 0.007 |
|  | Tu et al, 2018[43] | 30 | = 0.24 | | Fixed | 0.34 [0.20, 0.58] | 3.99 | ＜0.0001 |
|  | Tu et al, 2019[44] | 49 | = 0.14 | | Fixed | 0.36 [0.21, 0.62] | 3.63 | ＜0.0003 |
| PON | Amir et al, 2007[47] | 47 | = 0.04 | | Fixed | 0.54 [0.46, 0.63] | 7.62 | ＜0.00001 |
|  | Chen et al, 1998[35] | 43 | = 0.06 | | Fixed | 0.51 [0.43, 0.60] | 7.83 | ＜0.00001 |
|  | Chen et al, 2016[48] | 49 | = 0.03 | | Fixed | 0.53 [0.45, 0.63] | 7.68 | ＜0.00001 |
|  | Christensen et al, 1989[41] | 49 | = 0.03 | | Fixed | 0.53 [0.45, 0.62] | 7.90 | ＜0.00001 |
|  | Li et al, 2017[45] | 51 | = 0.02 | | Fixed | 0.53 [0.45, 0.61] | 8.05 | ＜0.00001 |
|  | Liu et al, 2008[36] | 45 | = 0.05 | | Fixed | 0.54 [0.46, 0.64] | 7.28 | ＜0.00001 |
|  | Liu et al, 2015[51] | 44 | = 0.05 | | Fixed | 0.50 [0.43, 0.59] | 8.50 | ＜0.00001 |
|  | Rusy et al, 2002[38] | 47 | = 0.04 | | Fixed | 0.51 [0.42, 0.60] | 7.46 | ＜0.00001 |
|  | Yang et al, 2015[49] | 51 | = 0.02 | | Fixed | 0.52 [0.44, 0.62] | 7.80 | ＜0.00001 |
|  | Yao et al, 2015[54] | 50 | = 0.03 | | Fixed | 0.51 [0.43, 0.60] | 7.83 | ＜0.00001 |
|  | Ye et al, 2008[34] | 51 | = 0.02 | | Fixed | 0.53 [0.45, 0.61] | 8.05 | ＜0.00001 |
|  | Zheng et al, 2008[55] | 50 | = 0.03 | | Fixed | 0.53 [0.45, 0.62] | 7.80 | ＜0.00001 |
|  | Zhang et al, 2014[42] | 7 | = 0.38 | | Fixed | 0.57 [0.49, 0.67] | 6.87 | ＜0.00001 |
| POV | Amir et al, 2007[47] | 16 | = 0.29 | | Fixed | 0.62 [0.47, 0.82] | 3.38 | = 0.0007 |
|  | Chen et al, 1998[35] | 35 | = 0.12 | | Fixed | 0.55 [0.39, 0.76] | 3.58 | = 0.0003 |
|  | Chen et al, 2016[48] | 35 | = 0.12 | | Fixed | 0.55 [0.40, 0.76] | 3.63 | = 0.0003 |
|  | Li et al, 2017[45] | 25 | = 0.21 | | Fixed | 0.58 [0.44, 0.78] | 3.69 | = 0.0002 |
|  | Liu et al, 2008[36] | 35 | = 0.12 | | Fixed | 0.55 [0.40, 0.76] | 3.63 | = 0.0003 |
|  | Liu et al, 2015[51] | 29 | = 0.17 | | Fixed | 0.52 [0.38, 0.72] | 4.03 | ＜0.00001 |
|  | Rusy et al, 2002[38] | 0 | = 0.54 | | Fixed | 0.50 [0.37, 0.67] | 4.60 | ＜0.0001 |
|  | Yang et al, 2015[49] | 36 | = 0.11 | | Fixed | 0.53 [0.38, 0.75] | 3.65 | = 0.0003 |
|  | Yao et al, 2015[54] | 24 | = 0.22 | | Fixed | 0.60 [0.44, 0.81] | 3.33 | = 0.0009 |
|  | Ye et al, 2008[34] | 35 | = 0.12 | | Fixed | 0.56 [0.41, 0.76] | 3.66 | = 0.0002 |
|  | Zheng et al, 2008[55] | 26 | = 0.20 | | Fixed | 0.59 [0.43, 0.80] | 3.42 | = 0.0006 |
|  | Zhang et al, 2014[42] | 28 | = 0.18 | | Fixed | 0.58 [0.43, 0.79] | 3.48 | = 0.0005 |
| Numbers needing antiemetic rescue | Amir et al, 2007[47] | 48 | = 0.06 | | Random | 0.64 [0.45, 0.92] | 2.42 | = 0.02 |
|  | Chen et al, 1998[35] | 42 | = 0.10 | | Random | 0.54 [0.38, 0.77] | 3.44 | = 0.0006 |
|  | Kabalak et al, 2005[32] | 57 | = 0.02 | | Random | 0.60 [0.42, 0.86] | 2.77 | = 0.006 |
|  | Liu et al, 2008[36] | 48 | = 0.06 | | Random | 0.64 [0.45, 0.92] | 2.42 | = 0.02 |
|  | Rusy et al, 2002[38] | 33 | = 0.17 | | Random | 0.54 [0.38, 0.77] | 3.47 | = 0.0005 |
|  | Yang et al, 2015[49] | 57 | = 0.02 | | Random | 0.59 [0.40, 0.86] | 2.75 | = 0.006 |
|  | Yeoh et al, 2016[31] | 57 | = 0.02 | | Random | 0.59 [0.40, 0.88] | 2.58 | = 0.010 |
|  | Yu et al, 2020[50] | 53 | = 0.04 | | Random | 0.62 [0.43, 0.91] | 2.46 | = 0.01 |
|  | Zheng et al, 2008[55] | 50 | = 0.05 | | Random | 0.64 [0.45, 0.91] | 2.50 | = 0.01 |
| Adverse Events | Amir et al, 2007[47] | 48 | = 0.06 | | Fixed | 0.86 [0.56, 1.31] | 0.71 | = 0.48 |
|  | Chen et al, 1998[35] | 56 | = 0.03 | | Fixed | 1.04 [0.61, 1.77] | 0.13 | = 0.90 |
|  | Kabalak et al, 2005[32] | 51 | = 0.05 | | Fixed | 0.89 [0.58, 1.35] | 0.57 | = 0.57 |
|  | Li et al, 2017[45] | 54 | = 0.03 | | Fixed | 0.91 [0.60, 1.39] | 0.43 | = 0.67 |
|  | Tu et al, 2019[44] | 52 | = 0.04 | | Fixed | 0.88 [0.58, 1.35] | 0.57 | = 0.57 |
|  | wang et al, 2014[56] | 46 | = 0.07 | | Fixed | 1.24 [0.77, 1.98] | 0.88 | = 0.38 |
|  | Yang et al, 2015[49] | 39 | = 0.12 | | Fixed | 0.78 [0.50, 1.19] | 1.15 | = 0.25 |
|  | Zhang et al, 2014[42] | 49 | = 0.05 | | Fixed | 1.14 [0.73, 1.77] | 0.58 | = 0.56 |
|  | Zárate et al, 2001[33] | 54 | = 0.03 | | Fixed | 0.91 [0.60, 1.38] | 0.43 | = 0.67 |

Note: RR, risk ratio; PONV, postoperative nausea and vomiting; PON, postoperative nausea; POV, postoperative vomiting.
