# Supplementary material for: Efficacy and safety of electrical acupoint stimulation for postoperative nausea and vomiting: A systematic review and meta-analysis
Source: PLoS One. 2023 May 31;18(5):e0285943. doi: 10.1371/journal.pone.0285943 (PMC10231798; doi:10.1371/journal.pone.0285943)
Supplement: S4 Table — (DOCX) [file pone.0285943.s010.docx]

**S4 Table.** Sensitivity analysis by changing the effects model for outcome analysis.

| Outcomes of interest | RR | Random effect model | Fixed effect model |
| --- | --- | --- | --- |
| PONV |  |  |  |
| PONV within 24 h | RR | 0.54 [0.46, 0.64] | 0.51 [0.43, 0.60] |
| PONV at other times | RR | 0.43 [0.24, 0.76] | 0.40 [0.25, 0.64] |
| PON | RR | 0.54 [0.43, 0.68] | 0.53 [0.45, 0.62] |
| POV | RR | 0.56 [0.42, 0.76] | 0.56 [0.45, 0.70] |
| Numbers needing antiemetic rescue | RR | 0.60 [0.43, 0.85] | 0.62 [0.49, 0.77] |
| Adverse effects | RR | 1.13 [0.49, 2.62] | 0.94 [0.62, 1.42] |

Note: RR, risk ratio; PONV, postoperative nausea and vomiting; PON, postoperative nausea; POV, postoperative vomiting.
