# Supplementary material for: Efficacy and safety of electrical acupoint stimulation for postoperative nausea and vomiting: A systematic review and meta-analysis
Source: PLoS One. 2023 May 31;18(5):e0285943. doi: 10.1371/journal.pone.0285943 (PMC10231798; doi:10.1371/journal.pone.0285943)
Supplement: Attachment — Submitted filename: Response to Reviewers.docx [file pone.0285943.s013.docx]

General comments:

We thank the editor and reviewers for their helpful suggestions and we have addressed all the points raised in our revised manuscript, and the revisions are outlined below. In addition to addressing these comments, we have also carefully reviewed the manuscript throughout to ensure clarity and reduce redundancy and repetition, and comply with Nature Communications style and formatting.

Editor and Reviewer comments:

Reviewer #1: Efficacy and safety of electrical acupoint stimulation for postoperative nausea and vomiting: A systematic review and meta-analysis
Thank you for the opportunity to review this submission to the journal. I have a small number of comments.

1. The submission is similar to the meta-analysis of Chen (Chen J, Tu Q, Miao S, Zhou Z, Hu S. Transcutaneous electrical acupoint stimulation for preventing postoperative nausea and vomiting after general anesthesia: a meta-analysis of randomized controlled trials. International Journal of Surgery. 2020 Jan 1; 73: 57-64.) but has a more up to date search (up to March 2020 vs July 2019) and as expected has more publications under analysis. I would have expected all of the studies included by Chen to be included in this submission but they are not – approximately half are included. What is the difference in the inclusion/exclusion criteria.

**Reply：**We appreciate your professional question. Due to the influence of anesthetic factors, type of surgery, and patient-related factors on PONV ^[1]^, we added 2 exclusion criteria compared to Chen's study: (1) Exclusion of patients with ASA ≥ III; (2) Excluding patients in the control group who received any electrical stimulation. That is why only about half of the studies included by Chen were included in our study. We’re sorry that the inaccurate description in the manuscript may lead to misunderstanding. We have revised the inclusion and exclusion criteria in the method accordingly (line 99 to 110). The reasons for excluding these articles are listed in the following table:

| Study | Points that do not meet our requirements | Our inclusion criteria |
| --- | --- | --- |
| Huang et al, 2017 | ASA 1/2/3 | ASA≤II |
| Wang et al, 2010 | Patients in the control group received electrical stimulation | No electrical stimulation in the control group |
| Xu et al, 2012 | ASA 1/2/3 | ASA≤II |
| Gan et al, 2004 | ASA 1/2/3 | ASA≤II |
| Kim et al, 2011 | In the control group，single twitch stimulation with 1 Hz was applied. | No electrical stimulation in the control group |
| Sun et al, 2017 | For sham group, patients were stimulated with the minimal current that the patient could feel. | No electrical stimulation in the control group |

Thank you again for your helpful question.

[1] Apfel CC, Roewer N. Risk assessment of postoperative nausea and vomiting. Int Anesthesiol Clin. 2003;41(4):13-32. Epub 2003/10/24. doi: 10.1097/00004311-200341040-00004.

2. After a brief search of the literature there were a number of publications that seem eligible for analysis such as those below. The second was published online in February 2020 and seems eligible.
H. Wang, Y. Xie, Q. Zhang, N. Xu, H. Zhong, H. Dong, L. Liu, T. Jiang, Q. Wang, L. Xiong, Transcutaneous electric acupoint stimulation reduces intra-operative remifentanil consumption and alleviates postoperative side-effects in patients undergoing sinusotomy: a prospective, randomized, placebo-controlled trial, BJA: British Journal of Anaesthesia, Volume 112, Issue 6, June 2014, Pages 1075–1082, https://doi.org/10.1093/bja/aeu001
Chen J, Zhang Y, Li X, Wan Y, Ji X, Wang W, Kang X, Yan W, Fan Z. Efficacy of transcutaneous electrical acupoint stimulation combined with general anesthesia for sedation and postoperative analgesia in minimally invasive lung cancer surgery: A randomized, double‐blind, placebo‐controlled trial. Thoracic cancer. 2020 Apr;11(4):928-34.
**Replay:** Thank you very much for this comment. Regarding the first literature (H. Wang, Y. Xie et al, 2014), we are very sorry that we excluded it by mistake. After rechecking and finding this to be an oversight on our part, we have revised all results and figures in the full paper. We are so sorry for our mistake.

Regarding the second study (Chen J, Zhang Y et al, 2020), we excluded it because patients in the sham-TEAS group received 4 mA stimulation. Our exclusion criteria included the control group receiving electrical stimulation.

Again, we thank the reviewer for pointing out this literature omission.

3. The findings are similar to that of Chen in terms of effect on PONV, nausea, vomiting and anti-emetic use. I would be interested to know what the authors suggestion is as to the proposed mechanism of effect. Is it simply an indirect effect through better analgesia and downstream opioid sparing which is emetogenic itself. Is it possible to deduce this from the data by comparing studies in which opioid consumption was measured.

**Replay:** Thanks for your suggestion. In deed, opioids have been long identified as an independent risk factor for the development of PONV ^[2]^. And evidences showed that EA treatment might lead to better analgesia and reducing opioid use. This is a possible mechanism of how EAS reduced PONV. However, it is hard to drop this conclusion because the amount of medication used was not explicitly given in a large part of the included literature. So we are sorry that can only add some discussion about the possible mechanism (line 405 to 409).

[2] Apfel CC, Laara E, Koivuranta M, Greim CA, Roewer N. A simplified risk score for predicting postoperative nausea and vomiting: conclusions from cross-validations between two centers. Anesthesiology. 1999;91(3):693–700. doi: 10.1097/00000542-199909000-00022.

4. Lastly is the anti-emetic effect more or less pronounced if anti-emetics are administered prophylactically. This would require an assessment of the anti-emetic interventions in the controls. I am not clear from the description of studies how the control groups were managed in terms of prophylaxis against PONV. It should be stated whether PONV prophylaxis was administered in the controls. Which studies were head to head RCTs of electroacupuncture vs anti-emetics and what was the meta-analytic result of these?
**Replay:** Thanks for your question. In the included studies, the control and treatment groups were consistent in their use of antiemetic prophylaxis. The prophylactic use of antiemetics is a routine practice after surgery even in the control group. In the literature we included, 7 explicitly stated the use of prophylactic antiemetics in the postoperative period, 4 stated that antiemetics were not used in the postoperative period, and the remaining 15 were not explicitly described in the literature.

And there were no head to head RCTs of electroacupuncture vs anti-emetics in our included studies.

5. Figures 2 and 3 are very blurred and hard to read in the main body of the document.

**Replay:** Thank you for pointing this out. We have re-uploaded the Figures in high resolution.

Reviewer #2: The paper entitled Efficacy and safety of electrical acupoint stimulation for postoperative nausea and vomiting: A systematic review and meta-analysis is well designed and well performed meta-analysis. The methods used here are appropriate, the manuscript is clear, well-organized. Only minor remarks are listed below:
- avoid using abbreviations in the abstract (TEAS)

**Replay:** We appreciate this valuable advice. We deleted all the abbreviations in the abstract.

- lines 74 and 77: repetition

**Replay:** We apologize for this oversight. This section has been modified to address this point (line 73 to 76).

-line 80: why not sufficiently comprehensive? Explain.

**Replay:** We are sorry that we didn’t describe this clearly. In our meta-analysis, we included more patients and RCTs. In addition to the inclusion of TEAS, RCTs evaluating the effectiveness of EA in the treatment of PONV were also added. What EA and TEAS have in common is that they both treat diseases by electrical acupoint stimulation. Thus, our study involves a more comprehensive analysis than the 2020 TEAS meta-analysis. We have moved this paragraph from the Introduction to the Discussion and modified the related description in the Discussion and changed the inappropriate statement (line 414 to 418).

- line 84: meanwhile is not the appropriate word.

**Replay:** We are sorry for the improper use of word. We moved this part to the Discussion and have revised the text (line 418).

- line 172: one category (3 articles) is missing

**Replay:** Thank you very much for pointing it out. In study selection, we added a category to describe three reports as published in abstract form (line 166).

- line 183: 7 or 8 trials?

**Replay:** Thank you for your great patience in listing the problems that exist with the manuscript. Here are 8 trials, we have added a Reference in the text (line 185).

- lines 233 AND 237: figure numbering to be corrected

**Replay:** Thank you for pointed out our mistake. We have modified the numbering of the figures (line 231 to 236).

Thank you again for your careful work.

Reviewer #3: Comments to the authors:
1. English language still needs some refinement and editing.

**Replay:** We thank the reviewer for the comment. The English language has been revised by an expert who is a native speaker.

2. Abbreviation used in the manuscript should have full forms at the time of first mentioning.

**Replay:** Thank you for pointing this out. We carefully reviewed the revision and made modifications. All of the abbreviations have full forms at the time of first mentioning now.

3. Some details should be discussed about assessment and severity of PONV using Apfel scoring system.

**Replay:** Thank you very much for your advice. We have added a paragraph in the discussion to describe in detail the assessment and severity of PONV using Apfel scoring system (line 397 to 409).

4. Not only the therapeutic measures, also commonly adopted preventive measures should also me mentioned.

**Replay:** Thank you so much. Routine use of 5-HT₃ receptor antagonists after surgery is the most common preventive measure. To address this point we added a sentence on the commonly adopted preventive measures in the clinic to the introduction (lines 60 to 65).

5. Many comments made about EAS are not supported by reference, which should be.

**Replay:** We agree with the reviewer and the reference has been added to the introduction. Thank you again for your helpful point.

6. Role of EAS outside China is questionable and thus not widely used, as claimed here, even now.

**Replay:** Thank you for pointing this out. we have removed this description from the introduction. We included 26 studies in 8 countries, so EA is described as increasingly popular in clinical.

7. Uniqueness of your meta-analysis compared to previous ones should be mentioned in the result and discussion sections, not in the introduction section.

**Replay:** Thanks for the suggestion and we have moved this part from Introduction to the Discussion section (line 410 to 421).

8. Did you use any filter during your literature search?

**Replay:** No, we did not use any filters when searching the literature. Thank you for your question.

9. What do you mean by “sham” treatment in the control group?

**Replay:** Compared to the treatment group, sham treatment in the control group included use of an inactive device, no needles with active device, patient-controlled analgesia + inactivated device, patient-controlled analgesia + usual care, which we corrected an inappropriate description in Table I and described the detail in the text (line 182 to 184; line 345 to 347).

10. Why were cesarean section and abortion excluded?

**Replay:** Thanks for your question. This is because the abortion patient may have pregnancy vomiting. Similarly, cesarean patients will use oxytocin. The oxytocin cause emesis-producing. And we included patients with general anesthesia, whereas most cesarean patients do not use this type of anesthesia. We describe this in Discussion to make this issue transparent (line 422 to 425).

11. Regarding the analysis of incidence of PONV, what was used in the control group? Also, you have mentioned incidence with EAS than presenting data separately on EA and TEAS.

**Replay:** Thank you for asking. The control group included in our study included: use of an inactive device, no needles with active device, no needles with inactive device, patient-controlled analgesia+ inactivated device, patient-controlled analgesia+ usual care. We have now revised this description in the text somewhat to be clearer (line 182 to 184; line 345 to 347). Our aim was to study the role of EAS, therefore, in describing the incidence of PONV, we used EAS as an overall intervention. In the fourth paragraph of the chapter, we did a subgroup analysis to determine whether EA or TEAS had treatment effect. The results showed that both EA and TEAS had significant therapeutic effects.

12. Did you do any subgroup analysis here between different types of surgery or not? Also was the anesthesia technique used standard in all the studies?

**Replay:** Thank you for your question. First, in our analysis, the surgical types, include laparoscopy, lithotripsy, tonsillectomy, thyroidectomy, gynecological, thoracotomy, craniotomy and plastic surgery. Thus, subgroup analysis according to surgery types will result in a small number of literature grouping.

Second, general anesthesia and standard anesthetic techniques was used in all the studies.

13. What do you mean by “EAS plus other treatments”? What are the other components of the other treatments? From the data I can see the additional other treatment did not decrease the incidence, rather increased the incidence of PONV a bit compared to only EAS. Why so?

**Replay:** Sorry for the undefined description. "EAS plus other treatments" means that there were other interventions in the treatment group in addition to EAS. Other treatments included the use of PCA in 6 articles and Dexameth in 1 article. Among these 7 studies, these other treatments were also applied in the control group. The variable between two groups in one article was the presence or absence of EAS. We have added an explanation in the "Incidence of PONV" section of our article.

14. In remaining 20% studies where you have mentioned that they received some treatment than placebo in the control arm, what are those interventions used?

**Replay:** Thank you for your helpful question. As for the control group, some studies used placebo treatment while others did not. Control patients in studies with no placebo treatment were given usual care, while those in studies with placebo treatment recieved either non-electric acupuncture or electrical stimulation to sham acupuncture points besides usual care. We have modified our text in the third part of the Result in order to clarify this point (line 231 to 236).

15. Apart from the incidence, did you do any analysis of frequency of PONV or not?

**Replay:** Thank you for your question. We did not do the analysis of frequency of PONV. This is an uncountable component due to the inclusion of very few studies documenting the PONV frequency. In deed, studies including PONV frequencies would make our analysis more credible.

16. Regarding timing of intervention used, not finding any difference between pre and postoperative EAS means, it does not have any better preemptive effect. Am I right? How do you explain this?

**Replay:** Thank you for your questions. Unfortunately, we cannot draw that conclusion, using the results obtained in our meta-analysis. Our analysis did not compare the effects of preoperative, intraoperative and postoperative EAS application. Because they are not in the same study. So we were unable to conclude which group had the best efficacy. And there are few such studies at present. In order to get this data, RCTs with higher quality are needed.

17. Targeted and comparative literature review is missing.
**Replay:** We sincerely appreciate the reviewer's insights. In the Discussion section, we have added a paragraph discussing the development of studies for meta-analysis related to our topic (line 411 to 422).

Strengths:

• Very relevant topic chosen for meta-analysis.
• Extensive literature search used.
• Extensive statistical analysis and subgroup analysis and multiple outcomes measured.

Weakness:

• English language used is very lucid, has several grammatical mistakes and lacked scientific tone.
• Some design flaws are quite obvious.
• No clarity on control or sham treatment group.
• No clear comparison with already recommended pharmacological treatment group.
• No obvious risk assessment or use of scoring system.
• Several repetitions of statistical methods in the methodology and result section. Especially the result section is confusing.
• The literature review is incomplete without targeted comparative analysis.

Verdict: Good intent of analyzing the efficacy of non-pharmacological measure in very relevant PONV which is still an intriguing issue despite abundance of antiemetics. But the manuscript still needs several modifications, refinement before I can accept it.
