# Supplementary material for: Efficacy and safety of electrical acupoint stimulation for postoperative nausea and vomiting: A systematic review and meta-analysis
Source: PLoS One. 2023 May 31;18(5):e0285943. doi: 10.1371/journal.pone.0285943 (PMC10231798; doi:10.1371/journal.pone.0285943)
Supplement: Attachment — Submitted filename: Response to Reviewers.docx [file pone.0285943.s014.docx]

Dear editor:

On behalf of my co-authors, thank you for giving us another chance to revise our article and improve its quality. We have read the comments carefully by you and other reviewers, and revisions have been done by now. Also, we have tried our best to revise our manuscript according to the comments. Attached please find the revised version, which we would like to submit for your kind consideration. Here, we would like to explain the changes briefly as follows:

1. Initial Question: English language still needs some refinement and editing.

My Reply: We thank the reviewer for the comment. The English language has been revised by an expert who is a native speaker.

Reviewers’ Reply: But I can see there are still many mistakes. Even your reply to my questions has several linguistic and grammatical mistakes. Either you take help from language expert or journal’s service.

**My New Response:** We apologize for the linguistic problems in the previous revision and my replies. We worked on the manuscript for a long time, and the repeated addition and removal of sentences obviously led to the previous polishing no longer being applicable. We now have asked American Journal Experts to help us with the language editing in this revision (certificate enclosed). We hope that the language in the current version of the manuscript is acceptable.

2. Initial Question: Abbreviation used in the manuscript should have full forms at the time of first mentioning.

My Reply: Thank you for pointing this out. We carefully reviewed the revision and made modifications. All of the abbreviations have full forms at the time of first mentioning now.

Reviewers’ Reply: Still many abbreviations are not mentioned in full form initially. Make sure when you reply that you have corrected, you have really address them.

**My New Response:** We apologize for the abbreviation issue. In this revision, all abbreviations were carefully checked to ensure that they are defined at first use. In addition, we have removed abbreviations that appear only once in our study and replaced them with the full wording. In addition, the abbreviations and their meanings are listed in the following table. Thank you for pointing this out.

| Abbreviation | Full words | Location |
| --- | --- | --- |
| PONV | Postoperative nausea and vomiting | line 51 |
| EA | Electroacupuncture | line 75 |
| TEAS | transcutaneous electrical acupoint stimulation | line 76 |
| EAS | electrical acupoint stimulation | line 78 |
| AMSTAR | Assessing the methodological quality of systematic reviews | line 94 |
| PON | postoperative nausea | line 114 |
| POV | postoperative vomiting | line 114 |
| RCT | randomized controlled trials | line 115 |
| ASA | American Society of Anesthesiologists | line 117 |
| GRADE | the Grades of Recommendation Assessment Development and Evaluation | line 149 |
| RR | relative risk | line 164 |
| CI | confidence interval | line 164 |

3. Initial Question: Some details should be discussed about assessment and severity of PONV using Apfel scoring system.

My Reply: Thank you very much for your advice. We have added a paragraph in the discussion to describe in detail the assessment and severity of PONV using Apfel scoring system (line 397 to 409).

Reviewers’ Reply: It should be done in the introduction or methodology, than in the discussion section. In the discussion section, regarding Apfel scoring, you only mentioned general part. Neither have discussed the pros and cons of that, nor have stressed why your own scoring system is superior to that one.

**My New Response:** Thank you so much for your questions regarding the scoring system. We apologize for not explaining more clearly why we did not use the Apfel scoring as the main observation in the previous version. The Apfel score is used to predict the risk of PONV but it does not show whether PONV occurred[1]. Our primary outcome index is the incidence of PONV, an objective indicator. Among the included studies, only one article used the Apfel scoring system. It specifies the inclusion of patients with an Apfel score ≥ 2 (the maximum score with this system is 4)[2]. This was done to include people with a high risk for PONV, while none of the other 25 studies had that inclusion criterion. Therefore, based on the current evidence base, we could not include Apfel scores in our analysis. However, we agree that the Apfel scoring system is very important. If these RCTs included Apfel scores in their inclusion criteria or stratified randomized groups of patients based on the Apfel scoring system, higher quality results would have been obtained. We have modified the discussion about Apfel scoring accordingly (lines 414 to 425).

When you mentioned our “scoring system”, were you referring to the GRADE system? GRADE is a well-established system used to assess the quality of evidence derived from RCTs. It classifies the evidence quality as A (high quality), B (medium quality), C (low quality), or D (very low quality)[3]. Its use is an important, highly recommended step in any comprehensive evidence evaluation and could increase the transparency of the decision-making process [4, 5]. If we misunderstood your question about scoring, please correct us. Thank you again for your helpful suggestion.

4. Initial Question: Not only the therapeutic measures, also commonly adopted preventive measures should also be mentioned.

My Reply: Thank you so much. Routine use of 5-HT₃ receptor antagonists after surgery is the most common preventive measure. To address this point we added a sentence on the commonly adopted preventive measures in the clinic to the introduction (lines 60 to 65).

Reviewers’ Reply: Do you think pharmacological mean is the only preventive measure, and nothing else need to be discussed here? Even your comment about routine use of 5-HT3 antagonist is not supported by evidence. You should know that drugs are not only the preventive and therapeutic means for managing PONV.

**My New Response:** We apologize for the insufficient clarity in our initial submission. As you mentioned, pharmacological treatment is not the only preventive and therapeutic means for managing PONV. We have added text describing nonpharmacological approaches to PONV prevention in the introduction (lines 68 to 72). To provide a better and more comprehensive description of the prevention and treatment of PONV, we have revised a paragraph in the introduction (lines 60 to 73). Thank you again for your helpful suggestion.

5. Initial Question: Many comments made about EAS are not supported by reference, which should be.

My Reply: We agree with the reviewer and the reference has been added to the introduction. Thank you again for your helpful point.

Reviewers’ Reply: Still I can see many comments are not supported by reference. Again, make sure what you reply here, actually you have done that in modified manuscript. No expert comment should be made in the manuscript without citing reference.

**My New Response:** Thank you very much for your careful review. We have carefully checked our manuscript and added 8 references (lines 59, 61, 62, 65, 77, 374 and 409). We hope there is no omission in the revised manuscript.

6. Initial Question: Role of EAS outside China is questionable and thus not widely used, as claimed here, even now.

My Reply: Thank you for pointing this out. we have removed this description from the introduction. We included 26 studies in 8 countries, so EA is described as increasingly popular in clinical.

Reviewers’ Reply: Use of EA in 8 countries does not mean universal acceptance. Moreover, can I know which are these 8 countries?

**My New Response:** The 26 studies included in our research were conducted in 8 countries: USA, The United Kingdom, China, India, Malaysia, Turkey, Iran, and Denmark. Table 1 lists the countries for each RCT in our manuscript. As you said, the use of EA in 8 countries does not mean universal acceptance. To make this clearer, we listed the names of these 8 countries in the results (lines 186 to 187). RCTs with higher quality and larger sample sizes need to be performed to promote the usage of EA worldwide. Thank you so much for the suggestion.

7. Initial Question: Uniqueness of your meta-analysis compared to previous ones should be mentioned in the result and discussion sections, not in the introduction section.

My Reply: Thanks for the suggestion and we have moved this part from Introduction to the Discussion section (line 410 to 421).

Reviewers’ Reply: Accepted.

**My New Response:** Thank you for accepting our reply.

8. Initial Question: Did you use any filter during your literature search?

My Reply: No, we did not use any filters when searching the literature. Thank you for your question.

Reviewers’ Reply: Then how you analysed the searched studies? Did you use only inclusion criteria then?

**My New Response:** We apologize for not answering your question clearly. We added more details to our search process, including the literature search strategy, inclusion criteria, and exclusion criteria.

In the first step, using the literature search strategy, we retrieved a total of 864 articles from the databases. In the second step, two researchers independently used EndNote reference management software for literature management. By screening the titles, abstracts, or both and removing duplicates, 794 records were excluded. In the third step, after reading the full articles, we excluded 44 RCTs that met the exclusion criteria. Finally, we included 26 studies.

Literature search strategy (Take PubMed as an example)

#1 Postoperative Nausea and Vomiting [Mesh]

#2 (“post operative” OR “postoperati*” OR “perioperati*” OR “peri-operative” OR “surger*” OR “surgical*” OR “postsurg*” OR “intraoperative” OR “anesthe*” OR “anaesthe*” OR “postanesthe*” OR “postanaesthe*” OR “anaesthetic recovery”)

#3 (“nause*” OR “vomit*” OR “emesis” OR “emeses” OR “emet*” OR “queasiness” OR “queasy”)

#4 #2 AND #3

#5 #1 OR #4

#6 Electroacupuncture [Mesh]

#7 electric*

#8 (acupuncture OR needle OR acupoint OR point OR stimulat*)

#9 #7 AND #8

#10 #6 OR #9

#11 Transcutaneous Electric Nerve Stimulation [Mesh]

#12 (“Transcutaneous Electrical Acupoint Stimulation” OR “TENS” OR “TEAS” OR “TNS” OR “ENS” OR “TES” OR “Transcutaneous electric* nerve stimulation” OR “transcutaneous nerve stimulation” OR “transcutaneous electric*” OR “transcutaneous electric* stimulation” OR “electric* nerve therap*” OR “electroanalgesi*” OR “electro-analgesi*” OR “Percutaneous Electric*” OR “Percutaneous Neuromodulation therap*” OR “Electroanalgesia*” OR “nerve stimulat*” OR “neuro-modulation” OR “neuromodulation” OR “neuromusc* electric*”)

#13 #11 OR #12

#14 Electric Stimulation Therapy [Mesh]

#15 (“Electric Stimulation Therapy” OR “Electric* Stimulation” OR “electrotherap*” OR “electrostimul*” OR “electromyostimulation” OR “Interferential Current Electrotherapy” OR “Therapeutic Electric* stimulat*” OR “Electric* stimulat* therap*”)

#16 #14 OR #15

#17 (“random* controlled trial” OR “random*” OR “placebo”)]

#18 #10 OR #13 OR #16

#19 #5 AND #18 AND #17

The inclusion criteria were:

1). Patients who underwent surgery, regardless of age, sex, ethnicity or surgery type

2). The intervention measures in the treatment group was EAS (EA or TEAS).

3). The control group could be sham acupuncture, placebo acupuncture, no treatment, nonacupoint acupuncture, no electrical stimulation or perioperative routine nursing.

4). The outcomes were the incidence of PONV, PON or POV.

5). To reduce the risk of bias and enhance the accuracy of the conclusions, only studies that had a randomized controlled trials (RCT) design were included.

The exclusion criteria were as follows:

1). Patients undergoing cesarean section or abortion.

2). American Society of Anesthesiologists ≥ III.

3). Patients in the control group who received any electrical stimulation.

4). Unpublished reports or reports published as abstracts only.

9. Initial Question: What do you mean by “sham” treatment in the control group?

My Reply: Compared to the treatment group, sham treatment in the control group included use of an inactive device, no needles with active device, patient-controlled analgesia + inactivated device, patient-controlled analgesia + usual care, which we corrected an inappropriate description in Table I and described the detail in the text (line 182 to 184; line 345 to 347).

Reviewers’ Reply: What do you mean by “inactive device”? Is it dry needling? That is also a treatment, isn’t it?

**My New Response:** We apologize for not clearly describing the different kinds of control treatments. The control treatments include the following four types:

When the treatment is EA, the control treatment includes: no needles + active device; no needles + inactive device or usual care,

When the treatment is TEAS, the control treatment includes: gel electrodes + inactive device or usual care,

- no needles + active device: The needles were bent to lay flat against the skin, or no needles were applied. Insulated wires from the activated stimulator box with a normal current output were attached to the needles or the inside of the arm covers.
- no needles + inactive device: The needles were bent to lay flat against the skin, or no needles were applied. Insulated wires from the inactivated stimulator box with no current output were attached to the needles or the inside of the arm covers.
- gel electrodes + inactive device: Gel electrodes were placed on the acupoints and attached to a stimulator box with no current output.

Therefore, “no needles + inactive device” is not a dry needling. It has no acupoint stimulation effect. It is a type of placebo. We performed subgroup analysis. The results showed that regardless of what was used as the control, placebo or usual care, EAS showed a significant role in reducing the incidence of PONV (lines 255 to 260). Therefore, we think “no needles + inactive device” should not be counted as a treatment, but only as a kind of psychological comfort.

10. Initial Question: Why were cesarean section and abortion excluded?

My Reply: Thanks for your question. This is because the abortion patient may have pregnancy vomiting. Similarly, cesarean patients will use oxytocin. The oxytocin cause emesis-producing. And we included patients with general anesthesia, whereas most cesarean patients do not use this type of anesthesia. We describe this in Discussion to make this issue transparent (line 422 to 425).

Reviewers’ Reply: Accepted.

**My New Response:** It’s very kind of you to accept our answer. Thank you so much.

11. Initial Question: Regarding the analysis of incidence of PONV, what was used in the control group? Also, you have mentioned incidence with EAS than presenting data separately on EA and TEAS.

My Reply: Thank you for asking. The control group included in our study included: use of an inactive device, no needles with active device, no needles with inactive device, patient-controlled analgesia+ inactivated device, patient-controlled analgesia+ usual care. We have now revised this description in the text somewhat to be clearer (line 182 to 184; line 345 to 347). Our aim was to study the role of EAS, therefore, in describing the incidence of PONV, we used EAS as an overall intervention. In the fourth paragraph of the chapter, we did a subgroup analysis to determine whether EA or TEAS had treatment effect. The results showed that both EA and TEAS had significant therapeutic effects.

Reviewers’ Reply: I am still very confused about inactive device with needle. Can you clarify this a bit more?

**My New Response:** We apologize for the confusion. In the answer to Question 9, we described all of the controls. The "inactive device with needle" you mentioned here should correspond to "no needle + inactive device", which means that the needles were bent to lay flat against the skin or no needles were applied and insulated wires from the activated stimulator box with no current output were attached to the needles or the insides of arm covers[6, 7].

We have revised this description in the text to make this more clear (lines 361 to 369).

12. Initial Question: Did you do any subgroup analysis here between different types of surgery or not? Also was the anesthesia technique used standard in all the studies?

My Reply: Thank you for your question. First, in our analysis, the surgical types, include laparoscopy, lithotripsy, tonsillectomy, thyroidectomy, gynecological, thoracotomy, craniotomy and plastic surgery. Thus, subgroup analysis according to surgery types will result in a small number of literature grouping.

Second, general anesthesia and standard anesthetic techniques was used in all the studies.

Reviewers’ Reply: Lithotripsy, thyroidectomy, thoracotomy, craniotomy, and plastic surgeries are not unique to have high PONV. I agree, that tonsillectomy, gynecological surgeries, laparoscopy are. But why squint surgery was omitted which is a prototype surgery to study PONV?

**My New Response:** Thank you for your professional question. Indeed, PONV is the most frequent complication in patients undergoing strabismus surgery[8]. We did not intentionally exclude squint surgery. A search of the literature revealed that these RCTs meeting the inclusion and exclusion criteria did not include squint surgery.

13. Initial Question: What do you mean by “EAS plus other treatments”? What are the other components of the other treatments? From the data I can see the additional other treatment did not decrease the incidence, rather increased the incidence of PONV a bit compared to only EAS. Why so?

My Reply: Sorry for the undefined description. "EAS plus other treatments" means that there were other interventions in the treatment group in addition to EAS. Other treatments included the use of PCA in 6 articles and Dexameth in 1 article. Among these 7 studies, these other treatments were also applied in the control group. The variable between two groups in one article was the presence or absence of EAS. We have added an explanation in the "Incidence of PONV" section of our article.

Reviewers’ Reply: Is PCA a treatment option for managing PONV? Dexamethasone has only preventive role in PONV, it is not a therapeutic option. I am really now confused about your open unfiltered search method.

**My New Response:** Thank you for raising this question. We defined PCA and dexamethasone as “other treatments” inappropriately. PCA is not a treatment option for managing PONV. It is a pain relief method given for postoperative analgesia. PCA is routinely used for postoperative analgesia in some countries. In addition, as you stated, dexamethasone is used as routine prophylaxis, not as a treatment. Six studies explicitly emphasized the use of PCA because all six studies were also about postoperative recovery or postoperative analgesia. One study explicitly emphasized the use of dexamethasone because it focused on the efficacy of dexamethasone and TEAS. Among these 7 studies, PCA/dexamethasone was also applied in the control group. Therefore, neither PCA nor dexamethasone is a specific treatment modality for PONV and is part of the standardized treatment regimen for both groups of patients. The attached table shows the 7 studies that emphasized the usage of PCA/dexamethasone. To remove the ambiguity, we have deleted the "EAS plus other treatments" from the table and text and removed the subgroup analysis of this topic from the results. Thank you again for the helpful feedback and comments on the manuscript.

| Author (year) | Title of the paper. |
| --- | --- |
|  |  |
| El-Rakshy et al, 2009 | Effect of intraoperative electroacupuncture on postoperative pain, analgesic requirements, nausea and sedation: a randomised controlled trial. |
| Gu et al, 2019 | The effect of pre-treatment with transcutaneous electrical acupoint stimulation on the quality of recovery after ambulatory breast surgery: a prospective, randomised controlled trial. |
| Chen et al, 2015 | ranscutaneous electric acupoint stimulation alleviates remifentanil-induced hyperalgesia in patients undergoing thyroidectomy: a randomized controlled trial. |
| Ye et al, 2008 | Pain management using Han's acupoint nerve stimulator combined with patient-controlled analgesia following neurosurgery: A randomized case control study |
| Chen et al, 1998 | The effect of location of transcutaneous electrical nerve stimulation on postoperative opioid analgesic requirement: acupoint versus nonacupoint stimulation. |
| Zheng et al, 2008 | Effect of transcutaneous electrical acupoint stimulation on nausea and vomiting induced by patient controlled intravenous analgesia with tramadol. |
| Yang et al, 2015 | Dexamethasone alone vs in combination with transcutaneous electrical acupoint stimulation or tropisetron for prevention of postoperative nausea and vomiting in gynaecological patients undergoing laparoscopic surgery. |

14. Initial Question: In remaining 20% studies where you have mentioned that they received some treatment than placebo in the control arm, what are those interventions used?

My Reply: Thank you for your helpful question. As for the control group, some studies used placebo treatment while others did not. Control patients in studies with no placebo treatment were given usual care, while those in studies with placebo treatment recieved either non-electric acupuncture or electrical stimulation to sham acupuncture points besides usual care. We have modified our text in the third part of the Result in order to clarify this point (line 231 to 236).

Reviewers’ Reply: Can placebo be called as a treatment option? I am more confused now.

**My New Response:** Except for usual care, the other control treatments can be considered a placebo. We performed subgroup analysis. The results showed that regardless of what was used as the control, placebo or usual care, EAS showed a significant role in reducing the incidence of PONV (lines 255 to 260). We think a placebo should not be counted as a treatment but only as a kind of psychological comfort.

The control treatments include the following four types:

When the treatment is EA, the control treatment includes: no needles + active device, no needles + inactive device or usual care.

When the treatment is TEAS, the control treatment includes: gel electrodes + inactive device and usual care.

15. Initial Question: Apart from the incidence, did you do any analysis of frequency of PONV or not?

My Reply: Thank you for your question. We did not do the analysis of frequency of PONV. This is an uncountable component due to the inclusion of very few studies documenting the PONV frequency. Indeed, studies including PONV frequencies would make our analysis more credible.

Reviewers’ Reply: Even if you have not done initially, you need to do it now as this is very important, and this you too have agreed.

**My New Response:** We apologize for not explaining more clearly why we did not perform an analysis of the frequency of PONV in the previous version of our manuscript. Only one of the 26 studies included in our meta-analysis recorded the frequency of PONV (please see the table below). Therefore, there was insufficient data to conduct a meta-analysis of the frequency of PONV. We have included the lack of data about the frequency of PONV as a limitation of this study (Discussion section lines 473 to 475). We hope this explains why we did not analyze the frequency of PONV.

| Author (year) | Number of participants  （TEAS / Control） | Frequency of PONV |
| --- | --- | --- |
| Yeoh et al, 2016 | 40/40 | No record |
| Kabalak et al, 2005 | 30/30 | TEAS group: 2 times;  Control group: 4 times |
| Zárate et al, 2001 | 110/56 | No record |
| Ye et al, 2008 | 20/20 | No record |
| Chen et al, 1998 | 25/25 | No record |
| Liu et al, 2008 | 48/48 | No record |
| An et al, 2014 | 41/40 | No record |
| Rusy et al, 2002 | 40/40 | No record |
| Sahmeddini et al, 2010 | 45/45 | No record |
| El-Rakshy et al, 2009 | 44/58 | No record |
| Christensen et al, 1989 | 10/10 | No record |
| Zhang et al, 2014 | 33/32 | No record |
| Tu et al, 2018 | 72/72 | No record |
| Tu et al, 2019 | 60/60 | No record |
| Li et al, 2017 | 20/20 | No record |
| Gu et al, 2019 | 58/59 | No record |
| Amir et al, 2007 | 20/20 | No record |
| Chen et al, 2016 | 46/46 | No record |
| Yang et al, 2015 | 50/50 | No record |
| Yu et al, 2020 | 30/30 | No record |
| Liu et al, 2015 | 44/44 | No record |
| Chen et al, 2015 | 29/30 | No record |
| Chen Y et al, 2015 | 41/42 | No record |
| Yao et al, 2015 | 35/36 | No record |
| Zheng et al, 2008 | 30/30 | No record |
| wang et al, 2014 | 30/30 | No record |

16. Initial Question: Regarding timing of intervention used, not finding any difference between pre and postoperative EAS means, it does not have any better pre-emptive effect. Am I right? How do you explain this?

My Reply: Thank you for your questions. Unfortunately, we cannot draw that conclusion, using the results obtained in our meta-analysis. Our analysis did not compare the effects of preoperative, intraoperative and postoperative EAS application. Because they are not in the same study. So we were unable to conclude which group had the best efficacy. And there are few such studies at present. In order to get this data, RCTs with higher quality are needed.

Reviewers’ Reply: When you did mention that you have done some subgroup analysis and that is the strength of your meta-analysis compared to the 2020 one, then you need to do this.

**My New Response:** We apologize for the unclear explanation in the previous version~~.~~ To reduce the incidence of PONV, the subgroup analysis showed that EAS, whether applied as a preoperative intervention, postoperative intervention or perioperative intervention, had a significant effect. We can only show that the three types of treatment timing are effective because they were not directly compared in any of the studies.

The sentence “Similarly, no significant subgroup differences were observed among the three types of treatment timing (RR, 0.51; 95% CI, 0.43 to 0.60; P = 0.33)” in the original text means that there was no significant difference in heterogeneity among the three subgroups. We apologize for the confusion. To avoid any further ambiguity, we have removed this sentence.

17. Initial Question: Targeted and comparative literature review is missing.

My Reply: We sincerely appreciate the reviewer's insights. In the Discussion section, we have added a paragraph discussing the development of studies for meta-analysis related to our topic (line 411 to 422).

Reviewers’ Reply: Targeted literature review does not only mean history of meta-analysis related to your topic and where yours one differ with previous one. This mean analysing the previous literature related to PONV, related to your areas of PONV topic and meta-analysis, and finally where they differed, why they differed from yours meta-analysis. Also, why yours one is unique and what new you have found from them. You have to defend your findings, and negate the differing findings of others.

**My New Response:** Thank you for the specific suggestion. we rewrote the literature review and analytic discussion section. We have added the targeted and comparative literature to the discussion accordingly (lines 438 to 464).

Thank you for giving us your constructive comments. We think that the revised manuscript has become more convincing.

Strengths:

• Very relevant topic chosen for meta-analysis.

• Extensive literature search used.

• Extensive statistical analysis and subgroup analysis and multiple outcomes measured.

Reviewers’ question: Why no comment here?

**My New Response:** We apologize for not responding to this comment last time. Thank you very much for your positive appraisal of the manuscript.

Weakness:

• English language used is very lucid, has several grammatical mistakes and lacked scientific tone.

• Some design flaws are quite obvious.

• No clarity on control or sham treatment group.

• No clear comparison with already recommended pharmacological treatment group.

• No obvious risk assessment or use of scoring system.

• Several repetitions of statistical methods in the methodology and result section. Especially the result section is confusing.

• The literature review is incomplete without targeted comparative analysis.

Reviewers’ question: Why no comment here?

**My New Response:** We apologize for not replying to your comments. Thank you for the reviewer’s careful reading of our manuscript. In light of all the above issues, we have carefully revised the document item by item.

Verdict: Good intent of analyzing the efficacy of non-pharmacological measure in very relevant PONV which is still an intriguing issue despite abundance of antiemetics. But the manuscript still needs several modifications, refinement before I can accept it.

Reviewers’ question: Why no comment here?

**My New Response:** We appreciate the reviewer’s succinct and accurate summary of our manuscript. Thank you for the kind statements. In light of your questions, we have carefully revised the document item by item. Thank you for taking the time to review our manuscript again. We hope that the current version of our manuscript reaches the standard for acceptance.

Reviewers’ Verdict: Although authors have tried to address the issues I have raised, but unfortunately, many issues still remain unaddressed even though authors have mentioned that they have done. Again considering the importance of the topic and the maturation of the manuscript, I want to give the authors another chance to readdress my points carefully. They should make sure that they have corrected the issues when they mention that they have done. Also they need to do some important subgroup analysis, PONV incidence comparison and should rewrite the literature review and analytic discussion section in a more scientific and argumentative way. If they come up with valid answers to my renewed points and reorganise their manuscript accordingly, I can give another re-look to the manuscript.

**My New Response:** Thank you for taking the time to read our revision and giving us your constructive comments. We have made the corresponding changes in the revised manuscript, and we think that the revised manuscript has become more rigorous and convincing. In the answer to the fifteenth question, we explained why we did not analyze the frequency of PONV. In addition, we rewrote the literature review and analytic discussion section. Thank you again for giving us the opportunity to have our manuscript reconsidered. We earnestly appreciate the reviewers’ hard work and hope that the corrections will meet with approval. Should you have any questions, please contact us without hesitation.

Reference

1.	Apfel CC, Greim CA, Haubitz I, Goepfert C, Usadel J, Sefrin P, et al. A risk score to predict the probability of postoperative vomiting in adults. Acta anaesthesiologica Scandinavica. 1998;42(5):495-501. Epub 1998/05/30. doi: 10.1111/j.1399-6576.1998.tb05157.x. PubMed PMID: 9605363.

2.	Yeoh AH, Tang SS, Abdul Manap N, Wan Mat WR, Said S, Che Hassan MR, et al. Effectiveness of P6 acupoint electrical stimulation in preventing postoperativenausea and vomiting following laparoscopic surgery. Turkish journal of medical sciences. 2016;46(3):620-5. Epub 2016/08/12. doi: 10.3906/sag-1502-56. PubMed PMID: 27513234.

3.	Balshem H, Helfand M, Schünemann HJ, Oxman AD, Kunz R, Brozek J, et al. GRADE guidelines: 3. Rating the quality of evidence. Journal of clinical epidemiology. 2011;64(4):401-6. Epub 2011/01/07. doi: 10.1016/j.jclinepi.2010.07.015. PubMed PMID: 21208779.

4.	Alonso-Coello P, Schünemann HJ, Moberg J, Brignardello-Petersen R, Akl EA, Davoli M, et al. GRADE Evidence to Decision (EtD) frameworks: a systematic and transparent approach to making well informed healthcare choices. 1: Introduction. BMJ (Clinical research ed). 2016;353:i2016. Epub 2016/06/30. doi: 10.1136/bmj.i2016. PubMed PMID: 27353417.

5.	Xin Z, Xue-Ting L, De-Ying K. GRADE in Systematic Reviews of Acupuncture for Stroke Rehabilitation: Recommendations based on High-Quality Evidence. Scientific reports. 2015;5:16582. Epub 2015/11/13. doi: 10.1038/srep16582. PubMed PMID: 26560971; PubMed Central PMCID: PMCPMC4642304.

6.	Rusy LM, Hoffman GM, Weisman SJ. Electroacupuncture prophylaxis of postoperative nausea and vomiting following pediatric tonsillectomy with or without adenoidectomy. Anesthesiology. 2002;96(2):300-5. Epub 2002/01/31. doi: 10.1097/00000542-200202000-00013. PubMed PMID: 11818760.

7.	Sahmeddini MA, Farbood A, Ghafaripuor S. Electro-acupuncture for pain relief after nasal septoplasty: a randomized controlled study. Journal of alternative and complementary medicine (New York, NY). 2010;16(1):53-7. Epub 2009/12/17. doi: 10.1089/acm.2009.0288. PubMed PMID: 20001536.

8.	Sayed JA, MA FR, MO MA. Comparison of dexamethasone or intravenous fluids or combination of both on postoperative nausea, vomiting and pain in pediatric strabismus surgery. Journal of clinical anesthesia. 2016;34:136-42. Epub 2016/10/01. doi: 10.1016/j.jclinane.2016.03.049. PubMed PMID: 27687360.
