# Supplementary material for: Efficacy and safety of electrical acupoint stimulation for postoperative nausea and vomiting: A systematic review and meta-analysis
Source: PLoS One. 2023 May 31;18(5):e0285943. doi: 10.1371/journal.pone.0285943 (PMC10231798; doi:10.1371/journal.pone.0285943)
Supplement: Attachment — Submitted filename: Response to Reviewers.docx [file pone.0285943.s015.docx]

Dear Editors and Reviewers:

Thank you very much again for giving us another chance to revise our article. And We are very grateful for your valuable suggestions and comments of the paper that are very helpful for us to improve our work. We have tried our best to revise our manuscript according to the comments. We are very happy that the reviewers accepted our explanation to some questions. We are now removing the questions that were accepted last time, leaving the questions that still need to be answered. We hope that your suggestions and comments could be perfectly addressed and the revised manuscript could meet the requirements for publication.

Attached please find the revised version, which we would like to submit for your kind consideration. We below present our responses and indicate where new information can be found in the original manuscript.

1. Initial Question: English language still needs some refinement and editing.

My Reply: We thank the reviewer for the comment. The English language has been revised by an expert who is a native speaker.

Reviewers’ Reply: But I can see there are still many mistakes. Even your reply to my questions has several linguistic and grammatical mistakes. Either you take help from language expert or journal’s service.

My last Response: We apologize for the linguistic problems in the previous revision and my replies. We worked on the manuscript for a long time, and the repeated addition and removal of sentences obviously led to the previous polishing no longer being applicable. We now have asked American Journal Experts to help us with the language editing in this revision (certificate enclosed). We hope that the language in the current version of the manuscript is acceptable.

Reviewers’ repeat response: I can still see several grammatical mistake, mistakes in sentence organisation as well as use of punctuation. I cannot do English editing repeatedly, and neither have time to do that. I leave this to the handling editor. If done properly, then OK for me.

**My New Response:** We thank you for the patience and suggestion. We have seriously revised our manuscript again. This has been done to the best of our abilities. We will appreciate if editor could help us to improve our manuscript writing.

3. Initial Question: Some details should be discussed about assessment and severity of PONV using Apfel scoring system.

My Reply: Thank you very much for your advice. We have added a paragraph in the discussion to describe in detail the assessment and severity of PONV using Apfel scoring system (line 397 to 409).

Reviewers’ Reply: It should be done in the introduction or methodology, than in the discussion section. In the discussion section, regarding Apfel scoring, you only mentioned general part. Neither have discussed the pros and cons of that, nor have stressed why your own scoring system is superior to that one.

My last Response: Thank you so much for your questions regarding the scoring system. We apologize for not explaining more clearly why we did not use the Apfel scoring as the main observation in the previous version. The Apfel score is used to predict the risk of PONV but it does not show whether PONV occurred[1]. Our primary outcome index is the incidence of PONV, an objective indicator. Among the included studies, only one article used the Apfel scoring system. It specifies the inclusion of patients with an Apfel score ≥ 2 (the maximum score with this system is 4)[2]. This was done to include people with a high risk for PONV, while none of the other 25 studies had that inclusion criterion. Therefore, based on the current evidence base, we could not include Apfel scores in our analysis. However, we agree that the Apfel scoring system is very important. If these RCTs included Apfel scores in their inclusion criteria or stratified randomized groups of patients based on the Apfel scoring system, higher quality results would have been obtained. We have modified the discussion about Apfel scoring accordingly (lines 414 to 425).

When you mentioned our “scoring system”, were you referring to the GRADE system? GRADE is a well-established system used to assess the quality of evidence derived from RCTs. It classifies the evidence quality as A (high quality), B (medium quality), C (low quality), or D (very low quality)[3]. Its use is an important, highly recommended step in any comprehensive evidence evaluation and could increase the transparency of the decision-making process [4, 5]. If we misunderstood your question about scoring, please correct us. Thank you again for your helpful suggestion.

Reviewers’ repeat response: Agree that Apfel scoring system identifies the at risk patients preoperatively, but it does predict the PONV as well. Although you have again mentioned the risk of PONV, but there is no mention how to evaluate and stratify those risks. This is confusing.

**My New Response:** Thank you again for your questions regarding the scoring system. In order to evaluate whether EAS is useful for patients with “low,” “medium,” or “high” risk of PONV, RCTs in which participants were stratified by Apfel score should be done. However, only one article mentioned the Apfel scoring system during patient inclusion among the included studies. It specifies the inclusion of patients with an Apfel score ≥ 2 (the maximum score with this system is 4) including patients with a medium-high risk for PONV [2]. This was done to include people with a medium or high risk for PONV, while none of the other 25 studies had that inclusion criterion. Thus, we could not conduct a subgroup analysis based on Apfel scores. We have added the discussion of how to evaluate and stratify the risk of PONV (Discussion section lines 454 to 468). And we have included the lack of scoring system as a limitation of this study (Discussion section lines 488 to 489).

4. Initial Question: Not only the therapeutic measures, also commonly adopted preventive measures should also be mentioned.

My Reply: Thank you so much. Routine use of 5-HT₃ receptor antagonists after surgery is the most common preventive measure. To address this point we added a sentence on the commonly adopted preventive measures in the clinic to the introduction (lines 60 to 65).

Reviewers’ Reply: Do you think pharmacological mean is the only preventive measure, and nothing else need to be discussed here? Even your comment about routine use of 5-HT3 antagonist is not supported by evidence. You should know that drugs are not only the preventive and therapeutic means for managing PONV.

My last Response: We apologize for the insufficient clarity in our initial submission. As you mentioned, pharmacological treatment is not the only preventive and therapeutic means for managing PONV. We have added text describing nonpharmacological approaches to PONV prevention in the introduction (lines 68 to 72). To provide a better and more comprehensive description of the prevention and treatment of PONV, we have revised a paragraph in the introduction (lines 60 to 73). Thank you again for your helpful suggestion.

Reviewers’ repeat response: Good for this acknowledgement. What I wanted to mean is that along with non-pharmacological means and modification of patient’s factors as well as the anaesthesia technique, using dexamethasone in the beginning and 5-HT3 antagonist at the end based on Apfel Risk score is the best method to prevent PONV. This should be clearly mentioned. Again in the non-pharmacological means you have mentioned crystalloids, chewing gum, ginger etc. These are indeed pharmacological means. Pharmacological means doesn’t have to me classic antiemetic medicine, but can be traditional medicines as well including IV fluids.

**My New Response:** Thank you very much for your precious reminding. According to your indication, we add the following sentence to the introduction section: Using dexamethasone in the beginning and 5-HT3 antagonist at the end based on Apfel Risk score is the best method to prevent PONV (Lines 64 to 66). As you pointed out, crystalloids, chewing gum, ginger are indeed pharmacological means. We made the correction (Lines 65 to 67; Lines 70 to 72).

8. Initial Question: Did you use any filter during your literature search?

My Reply: No, we did not use any filters when searching the literature. Thank you for your question.

Reviewers’ Reply: Then how you analysed the searched studies? Did you use only inclusion criteria then?

My last Response: We apologize for not answering your question clearly. We added more details to our search process, including the literature search strategy, inclusion criteria, and exclusion criteria.

In the first step, using the literature search strategy, we retrieved a total of 864 articles from the databases. In the second step, two researchers independently used EndNote reference management software for literature management. By screening the titles, abstracts, or both and removing duplicates, 794 records were excluded. In the third step, after reading the full articles, we excluded 44 RCTs that met the exclusion criteria. Finally, we included 26 studies.

Literature search strategy (Take PubMed as an example)

#1 Postoperative Nausea and Vomiting [Mesh]

#2 (“post operative” OR “postoperati*” OR “perioperati*” OR “peri-operative” OR “surger*” OR “surgical*” OR “postsurg*” OR “intraoperative” OR “anesthe*” OR “anaesthe*” OR “postanesthe*” OR “postanaesthe*” OR “anaesthetic recovery”)

#3 (“nause*” OR “vomit*” OR “emesis” OR “emeses” OR “emet*” OR “queasiness” OR “queasy”)

#4 #2 AND #3

#5 #1 OR #4

#6 Electroacupuncture [Mesh]

#7 electric*

#8 (acupuncture OR needle OR acupoint OR point OR stimulat*)

#9 #7 AND #8

#10 #6 OR #9

#11 Transcutaneous Electric Nerve Stimulation [Mesh]

#12 (“Transcutaneous Electrical Acupoint Stimulation” OR “TENS” OR “TEAS” OR “TNS” OR “ENS” OR “TES” OR “Transcutaneous electric* nerve stimulation” OR “transcutaneous nerve stimulation” OR “transcutaneous electric*” OR “transcutaneous electric* stimulation” OR “electric* nerve therap*” OR “electroanalgesi*” OR “electro-analgesi*” OR “Percutaneous Electric*” OR “Percutaneous Neuromodulation therap*” OR “Electroanalgesia*” OR “nerve stimulat*” OR “neuro-modulation” OR “neuromodulation” OR “neuromusc* electric*”)

#13 #11 OR #12

#14 Electric Stimulation Therapy [Mesh]

#15 (“Electric Stimulation Therapy” OR “Electric* Stimulation” OR “electrotherap*” OR “electrostimul*” OR “electromyostimulation” OR “Interferential Current Electrotherapy” OR “Therapeutic Electric* stimulat*” OR “Electric* stimulat* therap*”)

#16 #14 OR #15

#17 (“random* controlled trial” OR “random*” OR “placebo”)]

#18 #10 OR #13 OR #16

#19 #5 AND #18 AND #17

The inclusion criteria were:

1). Patients who underwent surgery, regardless of age, sex, ethnicity or surgery type

2). The intervention measures in the treatment group was EAS (EA or TEAS).

3). The control group could be sham acupuncture, placebo acupuncture, no treatment, nonacupoint acupuncture, no electrical stimulation or perioperative routine nursing.

4). The outcomes were the incidence of PONV, PON or POV.

5). To reduce the risk of bias and enhance the accuracy of the conclusions, only studies that had a randomized controlled trials (RCT) design were included.

The exclusion criteria were as follows:

1). Patients undergoing cesarean section or abortion.

2). American Society of Anesthesiologists ≥ III.

3). Patients in the control group who received any electrical stimulation.

4). Unpublished reports or reports published as abstracts only.

Reviewers’ repeat response: Accepted. The sentence of the inclusion and exclusion criteria should be revamped and organised to make it look more scientific.

**My New Response:** Thank you for your kind accepting and your suggestion. We have revamped and organized the inclusion and exclusion criteria (Lines 119 to 130).

The inclusion criteria were:

1. The review included emergency and elective surgery patients that underwent general anesthesia, regardless of age, sex, ethnicity, or surgery type.

2. The intervention measure in the treatment group was EAS (EA or TEAS).

3. the intervention measures in the control group were sham acupuncture, sham acupoint, sham electrical stimulation, or preoperative routine nursing.

4. Primary outcomes were the incidence of PONV, postoperative nausea (PON) or postoperative vomiting (POV). Secondary outcome measures were the numbers of patients requiring antiemetic rescue and adverse events.

5. The study was a randomized controlled trial (RCT) (blinded or non-blinded).

The exclusion criteria were as follows:

1. Patients undergoing cesarean section or abortion or those in the control group who received any electrical stimulation.

2. American Society of Anesthesiologists (ASA) ≥ III.

3. Literature with incorrect data or inaccessible data.

9. Initial Question: What do you mean by “sham” treatment in the control group?

My Reply: Compared to the treatment group, sham treatment in the control group included use of an inactive device, no needles with active device, patient-controlled analgesia + inactivated device, patient-controlled analgesia + usual care, which we corrected an inappropriate description in Table I and described the detail in the text (line 182 to 184; line 345 to 347).

Reviewers’ Reply: What do you mean by “inactive device”? Is it dry needling? That is also a treatment, isn’t it?

My New Response: We apologize for not clearly describing the different kinds of control treatments. The control treatments include the following four types:

When the treatment is EA, the control treatment includes: no needles + active device; no needles + inactive device or usual care,

When the treatment is TEAS, the control treatment includes: gel electrodes + inactive device or usual care,

- no needles + active device: The needles were bent to lay flat against the skin, or no needles were applied. Insulated wires from the activated stimulator box with a normal current output were attached to the needles or the inside of the arm covers.
- no needles + inactive device: The needles were bent to lay flat against the skin, or no needles were applied. Insulated wires from the inactivated stimulator box with no current output were attached to the needles or the inside of the arm covers.
- gel electrodes + inactive device: Gel electrodes were placed on the acupoints and attached to a stimulator box with no current output.

Therefore, “no needles + inactive device” is not a dry needling. It has no acupoint stimulation effect. It is a type of placebo. We performed subgroup analysis. The results showed that regardless of what was used as the control, placebo or usual care, EAS showed a significant role in reducing the incidence of PONV (lines 255 to 260). Therefore, we think “no needles + inactive device” should not be counted as a treatment, but only as a kind of psychological comfort.

Reviewers’ repeat response: I am a bit confused here. What did you exactly mean by “active” and “inactive” device with or without needle? In these complex and mixed methodology how did you do the double blinding?

**My New Response:** For the control treatments part, we are sorry for our confusing descriptions. Now, we have organized our mind so that we can be much easier to explain. In order to avoid any ambiguity, we rename “no needles” into “no acupuncture” (Lines: 106 to 107, 110, 216, and 271 to 272). The details of changes are as follows:

1. “active device” means Insulated wires from the activated stimulator box with a normal current output;

2.“Inactive device” means Insulated wires from the inactivated stimulator box with no current output (for example, the output wires of the stimulator were broken)
3. No needles essentially mean no acupuncture, include the following two types:

- with needle: The needles were bent to lay flat against the skin;
- without needle: the wire of the stimulator is pasted over the hand without needle and stimulation;

For the blinding part, we evaluated the reasonableness of the blinding method from the description of the 26 included studies. If the blinding was unclear, we marked it in yellow in our evaluation. If the blinding was not appropriate, we mark red in the evaluation (see S1 Fig). We apologize for not clearly describing the blinding. We think part of the articles are possible to be double blind, for the patients and outcome assessor. Considering the specificity of EAS treatment, blinding might not be done for operators (Lines 232 to 233).

11. Initial Question: Regarding the analysis of incidence of PONV, what was used in the control group? Also, you have mentioned incidence with EAS than presenting data separately on EA and TEAS.

My Reply: Thank you for asking. The control group included in our study included: use of an inactive device, no needles with active device, no needles with inactive device, patient-controlled analgesia+ inactivated device, patient-controlled analgesia+ usual care. We have now revised this description in the text somewhat to be clearer (line 182 to 184; line 345 to 347). Our aim was to study the role of EAS, therefore, in describing the incidence of PONV, we used EAS as an overall intervention. In the fourth paragraph of the chapter, we did a subgroup analysis to determine whether EA or TEAS had treatment effect. The results showed that both EA and TEAS had significant therapeutic effects.

Reviewers’ Reply: I am still very confused about inactive device with needle. Can you clarify this a bit more?

My Last Response: We apologize for the confusion. In the answer to Question 9, we described all of the controls. The "inactive device with needle" you mentioned here should correspond to "no needle + inactive device", which means that the needles were bent to lay flat against the skin or no needles were applied and insulated wires from the activated stimulator box with no current output were attached to the needles or the insides of arm covers[6, 7].

We have revised this description in the text to make this more clear (lines 361 to 369).

Reviewers’ repeat response: What is the active device without needle then?

**My New Response:** Thank you for your question. We are sorry for the unclear explanation. Active device without needle means no acupuncture, the activated stimulator box with a normal current output and the wire of the stimulator is pasted over the hand, without needle and stimulation. Hopefully our pictures will explain this.

13. Initial Question: What do you mean by “EAS plus other treatments”? What are the other components of the other treatments? From the data I can see the additional other treatment did not decrease the incidence, rather increased the incidence of PONV a bit compared to only EAS. Why so?

My Reply: Sorry for the undefined description. "EAS plus other treatments" means that there were other interventions in the treatment group in addition to EAS. Other treatments included the use of PCA in 6 articles and Dexameth in 1 article. Among these 7 studies, these other treatments were also applied in the control group. The variable between two groups in one article was the presence or absence of EAS. We have added an explanation in the "Incidence of PONV" section of our article.

Reviewers’ Reply: Is PCA a treatment option for managing PONV? Dexamethasone has only preventive role in PONV, it is not a therapeutic option. I am really now confused about your open unfiltered search method.

My Last Response: Thank you for raising this question. We defined PCA and dexamethasone as “other treatments” inappropriately. PCA is not a treatment option for managing PONV. It is a pain relief method given for postoperative analgesia. PCA is routinely used for postoperative analgesia in some countries. In addition, as you stated, dexamethasone is used as routine prophylaxis, not as a treatment. Six studies explicitly emphasized the use of PCA because all six studies were also about postoperative recovery or postoperative analgesia. One study explicitly emphasized the use of dexamethasone because it focused on the efficacy of dexamethasone and TEAS. Among these 7 studies, PCA/dexamethasone was also applied in the control group. Therefore, neither PCA nor dexamethasone is a specific treatment modality for PONV and is part of the standardized treatment regimen for both groups of patients. The attached table shows the 7 studies that emphasized the usage of PCA/dexamethasone. To remove the ambiguity, we have deleted the "EAS plus other treatments" from the table and text and removed the subgroup analysis of this topic from the results. Thank you again for the helpful feedback and comments on the manuscript.

| Author (year) | Title of the paper. |
| --- | --- |
|  |  |
| El-Rakshy et al, 2009 | Effect of intraoperative electroacupuncture on postoperative pain, analgesic requirements, nausea and sedation: a randomised controlled trial. |
| Gu et al, 2019 | The effect of pre-treatment with transcutaneous electrical acupoint stimulation on the quality of recovery after ambulatory breast surgery: a prospective, randomised controlled trial. |
| Chen et al, 2015 | ranscutaneous electric acupoint stimulation alleviates remifentanil-induced hyperalgesia in patients undergoing thyroidectomy: a randomized controlled trial. |
| Ye et al, 2008 | Pain management using Han's acupoint nerve stimulator combined with patient-controlled analgesia following neurosurgery: A randomized case control study |
| Chen et al, 1998 | The effect of location of transcutaneous electrical nerve stimulation on postoperative opioid analgesic requirement: acupoint versus nonacupoint stimulation. |
| Zheng et al, 2008 | Effect of transcutaneous electrical acupoint stimulation on nausea and vomiting induced by patient controlled intravenous analgesia with tramadol. |
| Yang et al, 2015 | Dexamethasone alone vs in combination with transcutaneous electrical acupoint stimulation or tropisetron for prevention of postoperative nausea and vomiting in gynaecological patients undergoing laparoscopic surgery. |

Reviewers’ repeat response: Partially agree. Even though your included studies have used PCA for postoperative analgesia (possibly opioid), it should not be included as measure for PONV, rather will be a proponent of causing PONV. Yes, I agree that prophylactic dexamethasone can really have in impact on the incidence of PONV and thus should be included in the meta-analysis.

**My New Response:** Thank you so much for your agreement.

17. Initial Question: Targeted and comparative literature review is missing.

My Reply: We sincerely appreciate the reviewer's insights. In the Discussion section, we have added a paragraph discussing the development of studies for meta-analysis related to our topic (line 411 to 422).

Reviewers’ Reply: Targeted literature review does not only mean history of meta-analysis related to your topic and where yours one differ with previous one. This mean analysing the previous literature related to PONV, related to your areas of PONV topic and meta-analysis, and finally where they differed, why they differed from yours meta-analysis. Also, why yours one is unique and what new you have found from them. You have to defend your findings, and negate the differing findings of others.

My Last Response: Thank you for the specific suggestion. we rewrote the literature review and analytic discussion section. We have added the targeted and comparative literature to the discussion accordingly (lines 438 to 464).

Thank you for giving us your constructive comments. We think that the revised manuscript has become more convincing.

Reviewers’ repeat response: Again I am unable to see targeted comparative literature review. For your every outcome measures you should mention some previous literature whether it is matching or you have found a new one. If not matching, why so. Simple descriptive literature review is unnecessary here.

General discussion on PONV and its etiology are immaterial here as this is very well-known. Keep the literature review pertaining to your topic of interest.

**My New Response:** Many thanks for your comments and suggestions. This time, discussion has been re-organized to introduce our outcome measures from the previous literature (Lines 373 to 427). For our outcome measures, we mention some previous literature (if similar literature exists). And we agree with you that the simple descriptive literature review is unnecessary here. So, we decided to delete the section. We hope you are satisfied with our revision, and thank you again for helping us improve the quality of this paper.

Weakness:

• English language used is very lucid, has several grammatical mistakes and lacked scientific tone.

• Some design flaws are quite obvious.

• No clarity on control or sham treatment group.

• No clear comparison with already recommended pharmacological treatment group.

• No obvious risk assessment or use of scoring system.

• Several repetitions of statistical methods in the methodology and result section. Especially the result section is confusing.

• The literature review is incomplete without targeted comparative analysis.

Reviewers’ question: Why no comment here?

My Last Response: We apologize for not replying to your comments. Thank you for the reviewer’s careful reading of our manuscript. In light of all the above issues, we have carefully revised the document item by item.

Reviewers’ repeat response: Your all loopholes and gaps need to be highlighted in the limitation section clearly.

Discussion should be started with findings of your meta-analysis than the group description. The description of groups with active or inactive with or without needles should come in the methodology or literature search section, not in the first paragraph of discussion.

**My New Response:** Thank you for pointing out those weakness of our manuscript. We sincerely apologize for all these shortcomings. In light of all the above issues, we have carefully revised the document item by item. For the issues (Some design flaws are quite obvious; No clear comparison with already recommended pharmacological treatment group; No obvious risk assessment or use of scoring system.) that could not be revised, we have listed them in the limitations (Lines 480 to 487).

We highly agree your comments. We moved the description of groups with active or inactive with or without needles to the literature search section. Thank you again for your advice (Lines 106 to 115).

Initial impression: Good intent of analyzing the efficacy of non-pharmacological measure in very relevant PONV which is still an intriguing issue despite abundance of antiemetics. But the manuscript still needs several modifications, refinement before I can accept it.

Reviewers’ question: Why no comment here?

My Last Response: Thank you for taking the time to read our revision and giving us your constructive comments. We have made the corresponding changes in the revised manuscript, and we think that the revised manuscript has become more rigorous and convincing. In the answer to the fifteenth question, we explained why we did not analyze the frequency of PONV. In addition, we rewrote the literature review and analytic discussion section. Thank you again for giving us the opportunity to have our manuscript reconsidered. We earnestly appreciate the reviewers’ hard work and hope that the corrections will meet with approval. Should you have any questions, please contact us without hesitation.

Reviewers’ Renewed Impression: Although the manuscript has matured much, but still there are areas which need to be improved further including the English language as well as targeted comparative literature review. I shall like to give authors another chance to improve the manuscript as it is improving in right direction. If they come up with clear explanations on the points I have raised and modify the manuscript accordingly I will give another look.

**My New Response:** We thank you for the positive comments and suggestions. We have tried our best to revise the manuscript according to your suggestions including grammatical mistake, mistakes in sentence organization as well as use of punctuation. As you say, we hope for help from the editor in grammar editing. Furthermore, the question about targeted comparative literature review, we rewrote the literature review and analytic discussion section. We have added the targeted and comparative literature to the discussion accordingly (Lines 373 to 427). For our outcome measures, we mention some previous literature. We hope this revision is appropriate. Thank you again for giving us the opportunity to revise our manuscript.

Reviewer #4:

1. Please describe the treatment methods, including the treatment site, treatment schedule, electrical stimulation intensity, evaluation schedule, and the results of RCTs included in this study in more detail.

My response: Thank you for your question and suggestion. We are happy to describe the treatment methods as your suggestions. We have added a Table 2 that include the treatment site, electrical stimulation intensity, and evaluation schedule. Treatment schedule and the results of RCTs included in this study had been describe in table 1, so we have not added these two to S2 Table. Thanks again to the reviewer for professional suggestions.

2. Please provide the literature search strategy of each electronic database as a additional file in a supporting information.

My response: Thank you for your advice. We have added the literature search strategy of each electronic database as an additional file in a supporting information (S1 Table). We hope that our modification is satisfactory to you.

3. Although the quality of evidence was moderate to low, the conclusion in the abstract section is too positive. Please revise it.

My response: Thank you for pointing this out. We agree with you that the conclusion in the abstract section is too positive. We have modified the sentences in abstract section (Lines 44 to 45). Again, thanks for this point.

4. The authors provided reference 32 and 33 as the basis for the sentence “ EAS is becoming increasingly popular in clinical practice due to its repeatability and standardization of frequency, intensity and duration “ in the introduction section ( page 4, line 82-83). I wonder how this sentence relates to reference 32 and 33.

**My response:** We are so sorry for the mistake. “EAS is becoming increasingly popular in clinical practice due to its repeatability and standardization of frequency, intensity and duration” in the introduction section is not objective. Therefor we delete this sentence including the reference (Line 83).

Reference

1.	Apfel CC, Greim CA, Haubitz I, Goepfert C, Usadel J, Sefrin P, et al. A risk score to predict the probability of postoperative vomiting in adults. Acta anaesthesiologica Scandinavica. 1998;42(5):495-501. Epub 1998/05/30. doi: 10.1111/j.1399-6576.1998.tb05157.x. PubMed PMID: 9605363.

2.	Yeoh AH, Tang SS, Abdul Manap N, Wan Mat WR, Said S, Che Hassan MR, et al. Effectiveness of P6 acupoint electrical stimulation in preventing postoperativenausea and vomiting following laparoscopic surgery. Turkish journal of medical sciences. 2016;46(3):620-5. Epub 2016/08/12. doi: 10.3906/sag-1502-56. PubMed PMID: 27513234.

3.	Balshem H, Helfand M, Schünemann HJ, Oxman AD, Kunz R, Brozek J, et al. GRADE guidelines: 3. Rating the quality of evidence. Journal of clinical epidemiology. 2011;64(4):401-6. Epub 2011/01/07. doi: 10.1016/j.jclinepi.2010.07.015. PubMed PMID: 21208779.

4.	Alonso-Coello P, Schünemann HJ, Moberg J, Brignardello-Petersen R, Akl EA, Davoli M, et al. GRADE Evidence to Decision (EtD) frameworks: a systematic and transparent approach to making well informed healthcare choices. 1: Introduction. BMJ (Clinical research ed). 2016;353:i2016. Epub 2016/06/30. doi: 10.1136/bmj.i2016. PubMed PMID: 27353417.

5.	Xin Z, Xue-Ting L, De-Ying K. GRADE in Systematic Reviews of Acupuncture for Stroke Rehabilitation: Recommendations based on High-Quality Evidence. Scientific reports. 2015;5:16582. Epub 2015/11/13. doi: 10.1038/srep16582. PubMed PMID: 26560971; PubMed Central PMCID: PMCPMC4642304.

6.	Rusy LM, Hoffman GM, Weisman SJ. Electroacupuncture prophylaxis of postoperative nausea and vomiting following pediatric tonsillectomy with or without adenoidectomy. Anesthesiology. 2002;96(2):300-5. Epub 2002/01/31. doi: 10.1097/00000542-200202000-00013. PubMed PMID: 11818760.

7.	Sahmeddini MA, Farbood A, Ghafaripuor S. Electro-acupuncture for pain relief after nasal septoplasty: a randomized controlled study. Journal of alternative and complementary medicine (New York, NY). 2010;16(1):53-7. Epub 2009/12/17. doi: 10.1089/acm.2009.0288. PubMed PMID: 20001536.

8.	Sayed JA, MA FR, MO MA. Comparison of dexamethasone or intravenous fluids or combination of both on postoperative nausea, vomiting and pain in pediatric strabismus surgery. Journal of clinical anesthesia. 2016;34:136-42. Epub 2016/10/01. doi: 10.1016/j.jclinane.2016.03.049. PubMed PMID: 27687360.
